# Use of paired Cas9-NG nickase and truncated sgRNAs for single-nucleotide microbial genome editing

**DOI:** 10.3389/fgeed.2024.1471720

**Published:** 2024-09-26

**Authors:** Song Hee Jeong, Ho Joung Lee, Sang Jun Lee

**Affiliations:** Department of Systems Biotechnology, Institute of Microbiomics, Chung-Ang University, Anseong, Republic of Korea

**Keywords:** paired nickase, Cas9-NG, truncated sgRNA, mismatch intolerance, single-nucleotide editing

## Abstract

The paired nickases approach, which utilizes clustered regularly interspaced short palindromic repeats (CRISPR)-CRISPR-associated proteins (Cas) nickase and dual guide RNA, has the advantage of reducing off-target effects by being able to double the target sequence. In this study, our research utilized the Cas9-NG nickase variant to minimize PAM sequence constraints, enabling the generation of paired nicks at desired genomic loci. We performed a systematic investigation into the formation sites for double nicks and the design of donor DNA within a bacterial model system. Although we successfully identified the conditions necessary for the effective formation of double nicks *in vivo*, achieving single-nucleotide level editing directly at the target sites in the genome proved challenging. Nonetheless, our experiments revealed that efficient editing at the single-nucleotide level was achievable on target DNA sequences that are hybridized with 5′-end-truncated dual single-guide RNAs (sgRNAs). Our findings contribute to a deeper understanding of the paired nickases approach, offering a single-mismatch intolerance design strategy for accurate nucleotide editing. This strategy not only enhances the precision of genome editing but also marks a significant step forward in the development of nickase-derived genome editing technologies.

## 1 Introduction

The CRISPR-Cas system, which was discovered as an adaptive immune system in prokaryotes, has been repurposed for genome editing. This modified CRISPR-Cas system consists of a Cas nuclease and guide RNA (gRNA), so it can specifically recognize and cleave target nucleic acid sequences. The gRNA/Cas nuclease complex induces double-strand breaks (DSBs) in the target DNA (Ran et al., 2013b). This DNA damage can be repaired by non-homologous end joining (NHEJ), which can lead to the loss of gene function through insertion and deletion (indel) mutations in the gene (Sander and Joung, 2014). If a donor DNA is added, the target DNA can be edited to the desired sequence through homology-directed repair (HDR) (Chu et al., 2015).

The CRISPR-Cas system was observed to cut similar sequences outside the desired target in the eukaryotic cell with high genome complexity (Lin et al., 2014). This off-target effect occurs due to the intrinsic characteristic of CRISPR-Cas that allows a mismatch between the target DNA and the spacer, and it has been considered as an obstacle to precise editing (Lee et al., 2020; Lin et al., 2014). Therefore, CRISPR-Cas nickase-based genome editing technologies have been developed to minimize DNA damage at undesired locations caused by off-target effects (Anzalone et al., 2020; Kim et al., 2024).

Cas9 nickase that generates single-strand breaks (nicks) in the target DNA can be made through either a D10A mutation in the RuvC domain or an H840A mutation in the HNH domain of Cas9 nuclease (Cong et al., 2013; Satomura et al., 2017). In the Cas9 nickase system, a pair of single-guide RNAs (sgRNAs) is utilized to create nicks on complementary strands at two adjacent DNA targets, thereby producing a DSB in the target DNA. Consequently, by doubling the length of the sequence recognizing the target, even if one sgRNA cleaves an off-target sequence, the absence of a nearby target sequence for a second sgRNA prevents DSBs, which reduces off-target editing (Shen et al., 2014). Specifically, it was reported that double nicking by paired sgRNAs in the Cas9 nickase system reduced off-target activity by up to 1,500-fold without decreasing target efficiency (Ran et al., 2013a). Based on these characteristics of nickase, Iriki et al. (2019) successfully performed a two-base substitution in the *Dictyostelium discoideum* genome.

The Cas9 nickase has been applied as a genome editing tool for various organisms, from microorganisms to humans (Liu et al., 2019; Roy et al., 2022; Standage-Beier et al., 2015; Klermund et al., 2024). Cas9(D10A) nickase enabled efficient deletion of target gene or gene clusters in *Streptomyces* sp. (Ma et al., 2023). Gopalappa et al. showed that the editing efficiency of paired Cas9 nickase for gene disruption via NHEJ in human and mouse cells is comparable to or higher than that of a Cas9 nuclease (Gopalappa et al., 2018). Additionally, it has been reported that the mutagenic targeting efficiency of Cas9(D10A) nickase is nine-fold higher than that of Cas9(H840A) nickase in human cells (Chiang et al., 2016). The nickase system is being utilized as a component in the development of base editor (BE) and prime editor (PE), which can edit the genome without forming DSB and introducing donor DNA. A BE, created by combining a base deaminase with Cas9(D10A) nickase, can induce C-to-T or A-to-G transition mutations in the target DNA (Gaudelli et al., 2017; Komor et al., 2016). However, BE can cause bystander editing, where C or A bases near the target C or A are also edited, because it deaminates the target base within a window of 4-5 nucleotides (nt) (Zhuang et al., 2022). To address this issue, a base editor with a narrow editing window was developed by fusing the truncated CDA1 deaminase domain to the Cas9(D10A) nickase (Tan et al., 2019). Additionally, a BE with reduced PAM restriction was developed by fusing the Cas9-NG nickase, which recognizes a 5′-NG PAM (Nishimasu et al., 2018), with PmCDA1-UGI (Zhong et al., 2019).

A PE, which consists of a reverse transcriptase fused to Cas9(H840A) nickase and a primer editing sgRNA (pegRNA), enables not only substitutions but also indels (Anzalone et al., 2020). Using a PE with Cas9(H840A) nickase and dual pegRNA, it was possible to insert a 1 kb DNA fragment or delete up to a 10 kb DNA fragment (Choi et al., 2022; Wang et al., 2022). Furthermore, a PE variant was developed by fusing the nickase of the near-PAMless SpCas9 variant, allowing mutations to be introduced into almost any region of the genome (Kweon et al., 2021; Walton et al., 2020). Recently, a new editing method called the click editor (CE), composed of a HUH endonuclease, a DNA-dependent DNA polymerase, and a nickase, has been developed to edit genomic target DNA according to the sequence of donor DNA (da Silva et al., 2024). While efforts to enhance editing efficiency and precision are ongoing, a notable limitation is that the large size of the constructs makes them difficult to deliver into cells via vectors (Jeong et al., 2023b). Consequently, there is a pressing need for research on HDR editing that employs only the Cas9 nickase motif. This approach minimizes damage to genomic DNA outside the target area.

In our study, we utilized the Cas9-NG nickase with 5′-NG as the PAM sequence (Nishimasu et al., 2018), which recognizes a shorter PAM sequence than wild type Cas9, reducing PAM sequence constraints and allowing recognition across the entire genome. We evaluated cell viability and genome editing efficiency in relation to the spacing of double nicks on the genome. We conducted a systematic examination in a microbial model system to observe the sequence alterations surrounding the editing site when the target sequence recognized by the sgRNA either overlaps or does not overlap with the editing location. Additionally, we explored sgRNA design strategies to address mismatch intolerance. Furthermore, we investigated approaches for designing donor DNA in genome editing utilizing nickase. We also discussed the mechanism and accuracy of nickase-mediated genome editing.

## 2 Materials and methods

### 2.1 Bacterial strains and culture conditions

Bacterial strains used in this study are listed in Supplementary Table S1. *Escherichia coli* was grown in LB broth (LPS solution, Korea, Cat. LB-05) at 30°C or 37°C, depending on the replication origin (*ori*) sequence of the plasmids. *E. coli* DH5α was used as a cloning host for the construction of dual sgRNA plasmids. To introduce the D10A mutation into the *cas9-NG* gene, the *E. coli* HK1159 strain carrying the *cas9-NG* gene located downstream of the L-arabinose-inducible P_*BAD*_ promoter in the chromosome of *E. coli* MG1655 was used. pHK463 (Kim et al., 2020), which expresses λ Bet protein, was used for the introduction of the D10A mutation by a single-strand oligonucleotide. *E. coli* HK1159 carrying pHK463 plasmid was cultured in LB broth containing ampicillin at 30°C. When the OD_600nm_ reached 0.4, L-arabinose (TCI, Japan, Cat. A0515) was added at a final concentration of 1 mM to overexpress Cas9-NG nuclease and λ red Bet protein. After 3 h, the cultured cells were harvested, washed twice with 10% glycerol, resuspended, and stored at −80°C. pHL143 sgRNA plasmid (200 ng) and D10A mutagenic oligonucleotide (100 pmol) were electroporated into the pHK463/HK1159 cells under conditions of 25 μF, 200 Ω, and 1.8 kV using a 0.1 cm electroporation cuvette (Bio-Rad, United States, Cat. 1652089). Afterward, the cells were transferred to 950 μL of SOC medium immediately and recovered for 1 h at 30°C and 180 rpm. Afterward, the cells were spread on LB agar containing spectinomycin and incubated for 16 h at 37°C. The successful incorporation of the D10A mutation into the *cas9-NG* gene was confirmed via Sanger sequencing. After the pHK463 plasmid was cured at 42°C, the strain was designated as *E. coli* SH169, in which the *cas9-NG(D10A)* gene was located downstream of the L-arabinose-inducible P_*BAD*_ promoter in the chromosome. If needed, kanamycin (25 μg/mL), ampicillin (50 μg/mL), and spectinomycin (75 μg/mL) were added to the medium.

### 2.2 Plasmid construction

The sgRNA plasmids used in this study are listed in Supplementary Table S1. Primers used for plasmid construction are listed in Supplementary Table S2. To generate nicks on different strands of the double-stranded target DNA, a plasmid was constructed that simultaneously expresses two sgRNAs targeting different strands within the *galK* gene (Supplementary Figure S1). We designed the plasmid to enable stable replication by positioning the spectinomycin resistance gene and the replication origin of the plasmid between the two sgRNAs. This arrangement prevents recombination between identical nucleotide sequences in the sgRNA scaffolds. A DNA fragment containing the first P_*j23119*_-sgRNA gene from pTargetF plasmid (Addgene #62226) and the spectinomycin resistance gene and another DNA fragment containing the second P_*j23119*_-sgRNA gene and *ori* sequence of the plasmids were amplified by PCR. PCR was performed using KOD FX (ToYoBo, Japan, Cat. KFX-101). Two purified DNA fragments were ligated using Gibson Assembly Master Mix (NEB, United Kingdom, Cat. E2611) and transformed into DH5α competent cells. The sequences of the dual sgRNA plasmids were confirmed through Sanger sequencing.

### 2.3 Genome editing

Mutagenic oligonucleotides (Supplementary Table S3) were synthesized to introduce quadruple-bases substitution (^494^TTGT → GATC, ^504^TAAC → ATCA, or ^528^AATT → GTAG) or single-base substitution (C490T, T504A, or C523T), generating a premature stop codon in the target *galK* gene. Mutagenic oligonucleotide (500 pmol) and dual sgRNA plasmid (200 ng) were electroporated into the *E. coli* SH169 cells carrying the pHK463 plasmid, in which both Cas9-NG(D10A) nickase and the λ red Bet protein were overexpressed in response to the addition of L-arabinose. As a negative control, pSR017 plasmid, which contains dual sgRNAs targeting the *galK* (498–517) and *xylB* (638–657) genes, was used (Lim et al., 2023). Electroporation was performed as mentioned above. The recovered cells were spread on MacConkey agar (BD Difco, United States, Cat. 281810) containing D-galactose (0.5%) (Samchun Chemicals, Korea, Cat. G0476) and spectinomycin, or LB agar containing spectinomycin, and then incubated for 16 h at 37°C.

For single-nucleotide editing using PCR products as donor DNA, double-stranded donor DNAs were amplified using PCR from a strain in which the C490T, T504A, or C523T mutation was introduced into the *galK* gene. Mutagenic PCR products of 1 kb size (500 ng) and 5′-end 2-nucleotide (nt)-truncated dual sgRNA plasmid (200 ng) were electroporated simultaneously into the SH169 cells carrying the pKD46 plasmid (Datsenko and Wanner, 2000). Electroporated cells were spread on MacConkey agar containing D-galactose (0.5%) and spectinomycin.

### 2.4 Two-step electroporation

Mutagenic oligonucleotide was synthesized to induce quadruple-bases substitution (^494^TTGT → GATC) in the *galK* gene, and the length of the oligonucleotides were 44mer, 67mer, 90mer, and 120mer. Mutagenic oligonucleotide (500 pmol) or pSH356 sgRNA plasmid (200 ng) was individually electroporated into L-arabinose-induced SH169 cells carrying the pHK463 plasmid and then incubated for 30 min at 37°C. Recovered cells were harvested, washed twice, and resuspended in a 10% glycerol solution. pSH356 sgRNA plasmid or mutagenic oligonucleotide was subsequently electroporated into each electrocompetent cell. After recovery for 30 min at 37°C, the cells were spread on MacConkey agar supplemented with D-galactose (0.5%) and spectinomycin.

### 2.5 Editing efficiency calculation

Genome editing efficiencies for the *galK* target were calculated by counting the number of red and white colonies in MacConkey agar [white colonies/(white colonies + red colonies)]. To determine whether the target was cleaved by the CRISPR-Cas nickase system, the number of surviving colonies in MacConkey agar supplemented with D-galactose (0.5%) and spectinomycin or LB agar supplemented with spectinomycin were counted. Colonies were randomly selected, and the edited DNA targets were analyzed by restriction enzyme and Sanger sequencing to confirm the accuracy of the Cas9-NG(D10A) nickase-mediated genome editing.

For restriction enzyme digestion analysis, a 1 kb DNA fragment containing the target region was amplified from white colonies randomly selected from MacConkey agar supplemented with D-galactose using galK-F and galK-R primers. PCR products were then incubated with BclI restriction enzyme (Enzynomics, Korea, Cat. R048S) for 90 min at 37°C. The digestion products were confirmed through 2% agarose gel electrophoresis. Primers used for PCR amplification and Sanger sequencing are listed in Supplementary Table S2.

### 2.6 *In vivo* nickase binding assay

The sgRNA plasmids targeting the promoter of the *xylA* gene in the xylose operon were constructed. Various sgRNA plasmids with different lengths of target recognition sequence (TRS) were transformed into the SH169 cells, as mentioned above. Each of the single colonies in LB agar supplemented with spectinomycin was streaked on MacConkey agar supplemented with D-xylose (0.3%) and spectinomycin. In addition, the transformant cells were streaked on MacConkey agar supplemented with L-arabinose (0.3%) and incubated at 37°C for 12 h. If sgRNA/Cas9-NG(D10A) nickase complex can repress the *xylA* promoter, and cells are not able to metabolize D-xylose, the color of colonies remains white. Conversely, if cells can utilize D-xylose due to a failure to repress the *xylA* promoter, the color turns red.

### 2.7 Statistical analysis

The data collected from experiments were analyzed on Microsoft Excel 2016 (Microsoft Corporation, United States) using two-tailed unpaired *t*-test to evaluate significance.

## 3 Results

### 3.1 The impact of the distance between double nicks on genome editing

**FIGURE 1:**
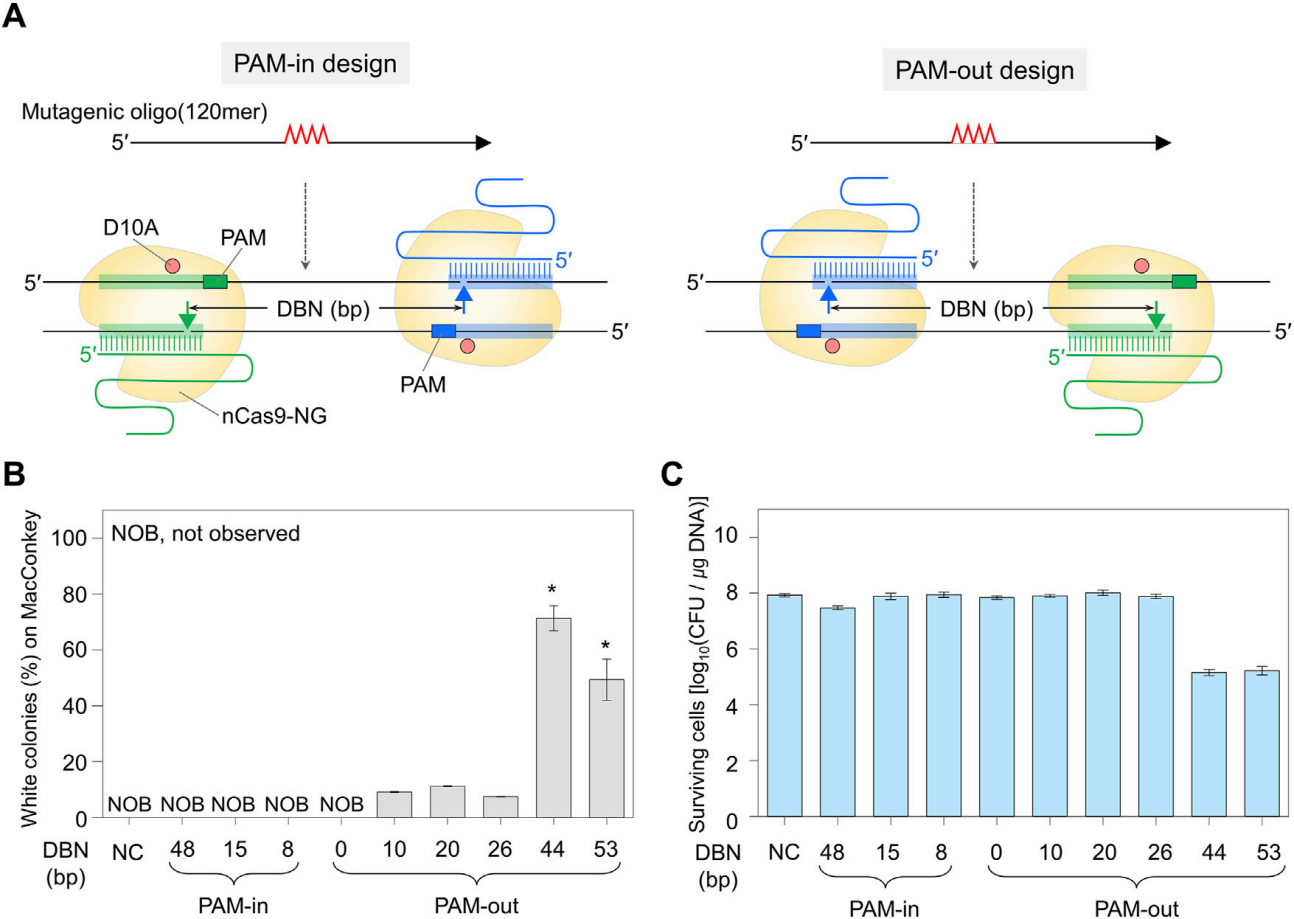
Double nick sites generated by Cas9-NG nickase and genome editing efficiency. **(A)** PAM-in and PAM-out designs differentiated by the relative positions of PAM and double nicks. Filled triangles indicate the nick sites formed by dual sgRNA/nickase complex. Mutagenic oligonucleotides can generate a quadruple mutation that contains a stop codon in the *galK* target. **(B)** Comparison of quadruple-base editing efficiency using PAM-in and PAM-out designs with various DBN. Editing efficiency (%) was calculated as the proportion of the white colonies to the total colonies formed on MacConkey agar containing D-galactose. *P*-values were determined by comparing the ratio of white colonies in DBN 44 and DBN 53 with those in DBN 0, 10, 20, and 26. *P < 0.05. Negative control (NC) used a dual sgRNA plasmid targeting the *galK* and *xylB* genes. **(C)** The number of surviving colonies depending on DBN. It indirectly indicates whether the Cas9-NG nickase complex could generate double nicks on the target DNA and cause cell death. Each bar represents the mean from three independent experiments. DBN stands for the distance between nicks.

We investigated the impact of the distance between nicks occurring around the editing site on the genome editing efficiency. A dual sgRNA plasmid was designed to have various distances between nicks (DBN), and a mutagenic oligonucleotide (120mer) was synthesized to introduce the four bases substitution (^504^TAAC → ATCA) in the *galK* gene. These constructs were electroporated into the *E. coli* SH169 cells overexpressing λ Bet protein and Cas9-NG nickase, a Cas9 nickase variant with expanded protospacer adjacent motif (PAM) (5′-NG) (Figure 1A; Supplementary Figure S2). When the target sequence in *galK* is properly altered by mutagenic oligonucleotides via the introduction of stop codons, the edited cells form white colonies in MacConkey agar containing D-galactose. Conversely, when the target was unedited, D-galactose can be normally metabolized, thereby forming red colonies (Supplementary Figure S3).

For the PAM-in design, in which the PAM sequence is located inside the double nicks, white colonies were not observed on MacConkey agar containing D-galactose, and the number of surviving colonies was high (≥10^7^ CFU/μg DNA). In contrast, for the PAM-out design, in which the PAM sequence is located outside the double nicks, white colonies were observed in the proportions of 9%, 11%, and 7% when the distances between the double nicks were 10, 20, and 26 base pairs (bp), respectively. When double nicks were formed 44 or 53 bp apart, the number of surviving colonies decreased significantly to 10^5^ CFU/μg DNA, and the proportion of white colonies was very high at 71% or 49%, respectively (Figures 1B, C). Quadruple-base substitutions in the *galK* target of randomly selected white colonies were confirmed through BclI restriction enzyme digestion and Sanger sequencing. As a result, for DBN 10, DBN 20, DBN 26, DBN 44, and DBN 53—where white colonies were observed—it was confirmed that the BclI restriction enzyme site had been inserted into the target sequence in four, four, four, two, and two out of four white colonies, respectively (Supplementary Figure S4).

Sanger sequencing revealed that all four white colonies were correctly edited in cases of overlapped targets (DBN 10, DBN 20, and DBN 26). However, in the case of DBN 44 and DBN 53, unwanted mutations were observed within the target sequence in all four colonies (Supplementary Figure S5). These outcomes suggest that while the probability of obtaining edited cells with high viability decreases when the bases to be edited are located on two overlapping target sequences (DBN 10, DBN 20, and DBN 26), the accuracy of the editing appears to be higher. Conversely, when the nucleotide sequence to be edited is positioned between targets, the target DNA is efficiently cleaved by the CRISPR-Cas9 nickase system, albeit with a decrease in editing accuracy. Additionally, these findings suggest that the outcomes of nickase-mediated genome editing cannot be conclusively verified using restriction enzymes. As a result, in subsequent experiments, the edited target sequences were analyzed through Sanger sequencing.

### 3.2 Effective genome editing by nickase-mediated negative selection

To enhance the genome editing accuracy under the PAM-out (DBN 44 bp) condition where target DNA is effectively cleaved, we examined how editing efficiency varies depending on the mutation locations. We designed mutations to be introduced on the target DNA hybridized with sgRNA or in between targets. Specifically, various lengths of mutagenic oligonucleotides (44mer, 67mer, 90mer, and 120mer) inducing four bases substitution at two mutagenesis sites (on Target1 and between Targets) were synthesized (Supplementary Figure S6). Each mutagenic oligonucleotide was electroporated along with a dual sgRNA plasmid with 44 bp DBN into cells overexpressing Cas9-NG(D10A) nickase and λ Bet protein.

**FIGURE 2:**
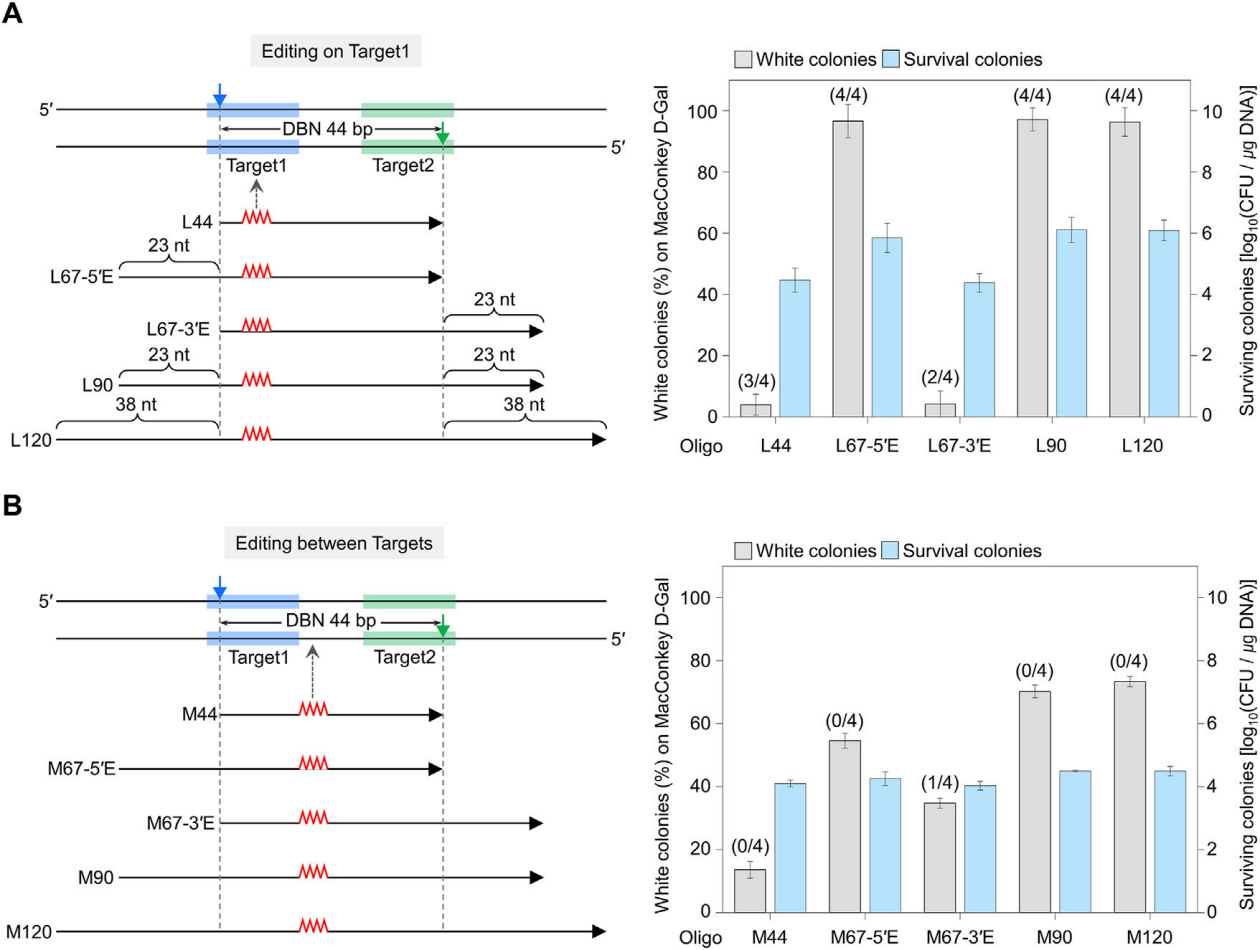
Comparison of editing efficiency where the sgRNA-target sequence overlaps with the editing site **(A)** and where the editing site does not overlap within the target sequence **(B)**. The blue- and green-shaded regions indicate the target DNA sequences recognized by each sgRNA. Filled triangles indicate the nick sites formed by the dual sgRNA/nickase complex. Mutagenic oligonucleotides with various lengths of the following homology arms were used: L44 has a length equivalent to the distance between nicks (DBN) of 44 bp. L67-5′E includes the DBN of 44 nt plus an extended homologous arm (23 nt) in the 5′ direction from the nick site (67 nt = 44 nt + 23 nt). L90 includes the DBN of 44 nt plus an extended homologous arm (23 nt) in both directions from the nick site (90 nt = 44 nt + 46 nt). L120 includes the DBN of 44 nt plus an extended homologous arm (38 nt) in both directions from the nick site (120 nt = 44 nt + 76 nt). Editing efficiency was calculated as the number of white colonies of the total (red + white) colonies. Editing accuracy was calculated based on the number of correctly edited colonies among white colonies, as confirmed by Sanger sequencing (as indicated in parentheses). Each bar represents the mean from three independent experiments.

In the case of On Target1, when L67-5′E, L90, and L120 were used, the percentage of white colonies was 96–97%, and almost all cells showed an edited phenotype. However, less than 5% of white colonies were obtained when L44 and L67-3′E were used (Figure 2A). In the case of Between Targets, when M67-5′E and M67-3′E were used, there was no significant difference in the proportion of white colonies, at 54% and 34%, respectively (Figure 2B). White colonies were randomly selected, and the nucleotide sequences of the edited *galK* targets were amplified and analyzed using Sanger sequencing (Supplementary Figure S7). When mutagenic oligos (L44, L67-5′E, L67-3′E, L90, and L120) that cause mutations in On Target1 were used, three, four, two, four and four out of four white colonies showed correct sequence editing, respectively. Conversely, when M67-3′E was used to induce mutations between targets, only one among four white colonies was correctly altered, while when M44, M67-5′E, M90, and M120 were used, all four white colonies showed unwanted mutations on the target DNA recognized by sgRNA. These results indicate that Cas9-NG nickase-mediated genome editing can be accurate when the nucleotide to be edited is located on the target sequence hybridized with sgRNA.

We investigated the genomic editing mechanism of the nickase system by employing a two-step electroporation method, which involves altering the order of electroporation for the dual sgRNA plasmid (pSH356) and the mutagenic oligonucleotides. As a result, when mutagenic oligos were electroporated first, followed by electroporation of pSH356 sgRNA plasmid, although the editing efficiency was lower than that achieved with simultaneous electroporation of both the oligo and sgRNA plasmid, the resultant patterns were similar (Figure 2A; Supplementary Figure S8A). However, when the sgRNA plasmid was electroporated first, white colonies were rarely obtained (Supplementary Figure S8B). These results indicate that oligonucleotides with homology arms are ineffective in facilitating the repair of cleaved chromosomes. When the target is cleaved, the majority of cells perish, making it impossible to obtain edited cells. Consequently, the results demonstrate that following mutagenesis, cells that have been edited can be successfully obtained via negative selection by employing the dual sgRNA/Cas9-NG nickase complex.

### 3.3 Genome editing efficiency according to PAM-out design

**FIGURE 3:**
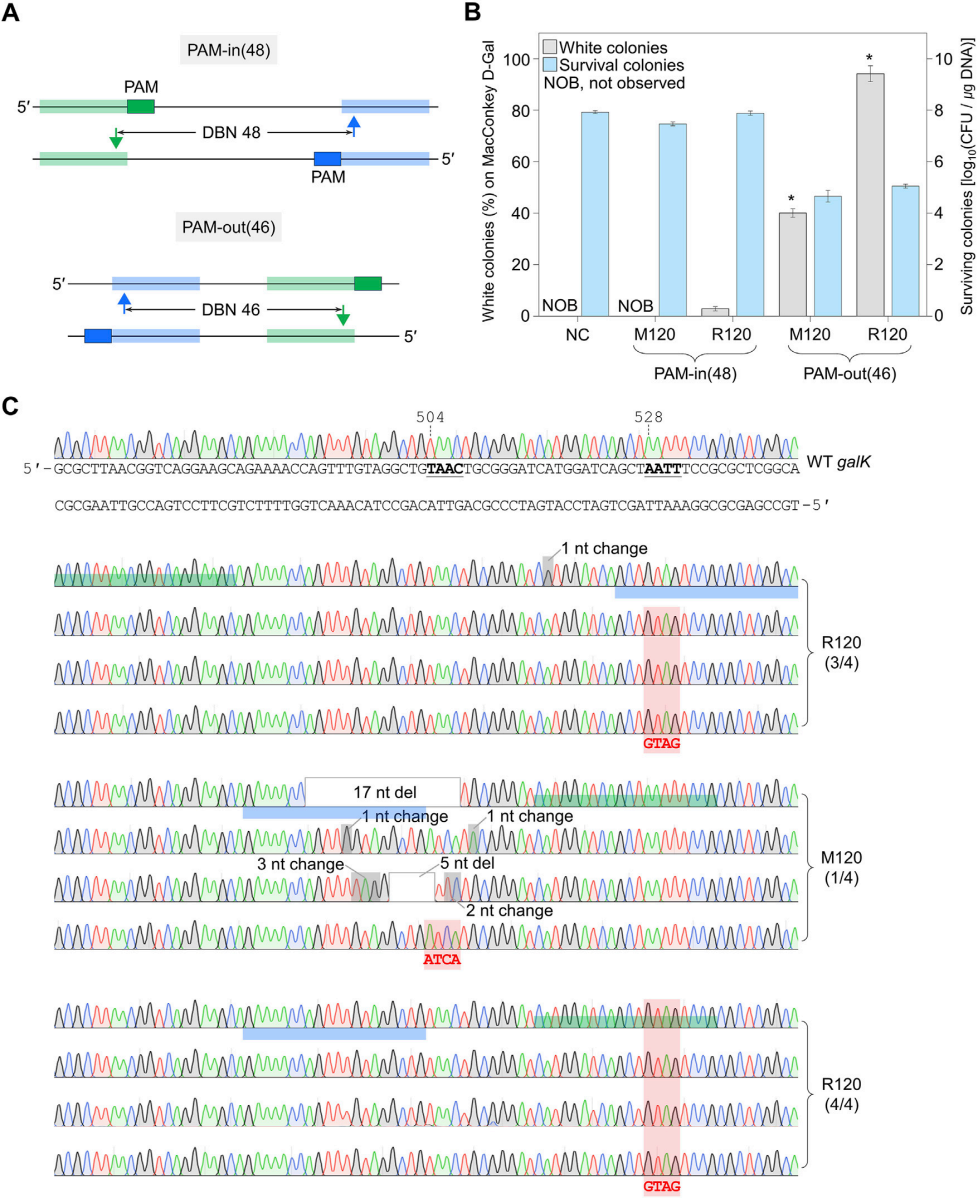
Genome editing efficiency of PAM-in and PAM-out designs. **(A)** PAM-in and PAM-out designs with similar distances between nicks. Blue- and green-shaded regions indicate the target DNA sequences recognized by each sgRNA. Filled triangles represent the positions where nicks are generated. **(B)** Efficiency of quadruple-base editing and the number of surviving colonies in *galK* target using sgRNAs with PAM-in and PAM-out designs. Editing efficiency was assessed by counting the white colonies on MacConkey agar plates supplemented with D-galactose. *P*-values were determined by comparing the ratio of white colonies between PAM-in (48) and PAM-out (46), both of which used the same mutagenic oligonucleotide. *P < 0.05. Negative control (NC) used a dual sgRNA plasmid targeting the *galK* and *xylB* genes. **(C)** Sanger sequences of quadruple-base-edited cells. Underlined bold letters indicate the target nucleotide for the genome editing. Blue- and green-shaded chromatograms are target sequences that are hybridized with each sgRNA. Red-shaded chromatograms indicate correctly edited bases (^504^TAAC → ATCA, ^528^AATT → GTAG). Gray boxes and gray-shaded chromatograms indicate unwanted mutations. Each bar represents the mean from three independent experiments. Numbers in parentheses are correctly edited colonies among white colonies selected for Sanger sequencing.

When PAM-in design sgRNA plasmid with a distance between double nicks at 48 bp apart was used, genome editing was not successful, and the number of survival colonies was increased, even if the DBN was sufficiently far (Figures 1B, C). To examine whether the PAM-in or PAM-out design, as well as the DBN, affects editing efficiency, we designed the PAM-out (46), which generates double nicks at a position similar to PAM-in (48) (Figure 3A; Supplementary Figure S9). In the case of PAM-in (48) dual sgRNA, when M120 oligo, which induces mutations (^504^TAAC → ATCA) between targets, was used, no white colony was formed, and the CFU was 10^6.9^/μg DNA. When R120 oligo, which induces mutations (^528^AATT → GTAG) on the target, was used, the percentage of white colonies was low, at 3%, and the number of surviving colonies was similar to that of M120. In the case of PAM-out (46) dual sgRNA, when M120 oligo was used, the percentage of white colonies was 40%, and the number of surviving colonies was decreased to 10^4.6^ CFU/μg DNA. When R120 oligo was used, the percentage of white colonies was remarkably increased to 94%, and the CFU was 10^5^/μg DNA (Figure 3B). Sanger sequencing was performed to confirm the editing accuracy (Figure 3C). When R120 oligo was used in PAM-in (48), three of the four white colonies showed correct sequence editing. In the case of PAM-out (46) using M120 oligo, only one among four white colonies was correctly edited. In contrast, with R120 oligo, only the desired mutation was introduced in all identified white colonies (Figure 3C). These results suggest that the PAM-out design effectively facilitates the formation of double nicks, and if base editing is designed on the target sequence hybridized by sgRNA, it is possible to achieve genome editing with significantly high efficiency.

### 3.4 Failure of accurate editing using untruncated sgRNA/nickase complex

We explored whether dual sgRNA with 20 nt-length sequences as TRS could be utilized for single-nucleotide editing by mutagenic oligonucleotide for each target at several mutagenesis sites. A mutagenic oligonucleotide that introduces a single-nucleotide substitution at three mutagenesis sites was electroporated with N_20_ dual sgRNA (DBN 44 bp) into both Cas9-NG(D10A) nickase- and λ Bet-overexpressing cells. The proportions of white colonies obtained using mutagenic oligonucleotides that induce mutations in On Target1, Between Targets, and On Target2 were 71%, 72%, and 70%, respectively (Supplementary Figure S10A). Subsequently, the edited nucleotide sequences were verified using Sanger sequencing. However, unwanted mutations were observed in all identified white colonies (Supplementary Figure S10B). These data indicate that the accuracy of single-nucleotide editing was very low when dual sgRNA with a 20 nt target sequence was used.

### 3.5 Maximally truncated sgRNA for *in vivo* nickase activity

**FIGURE 4:**
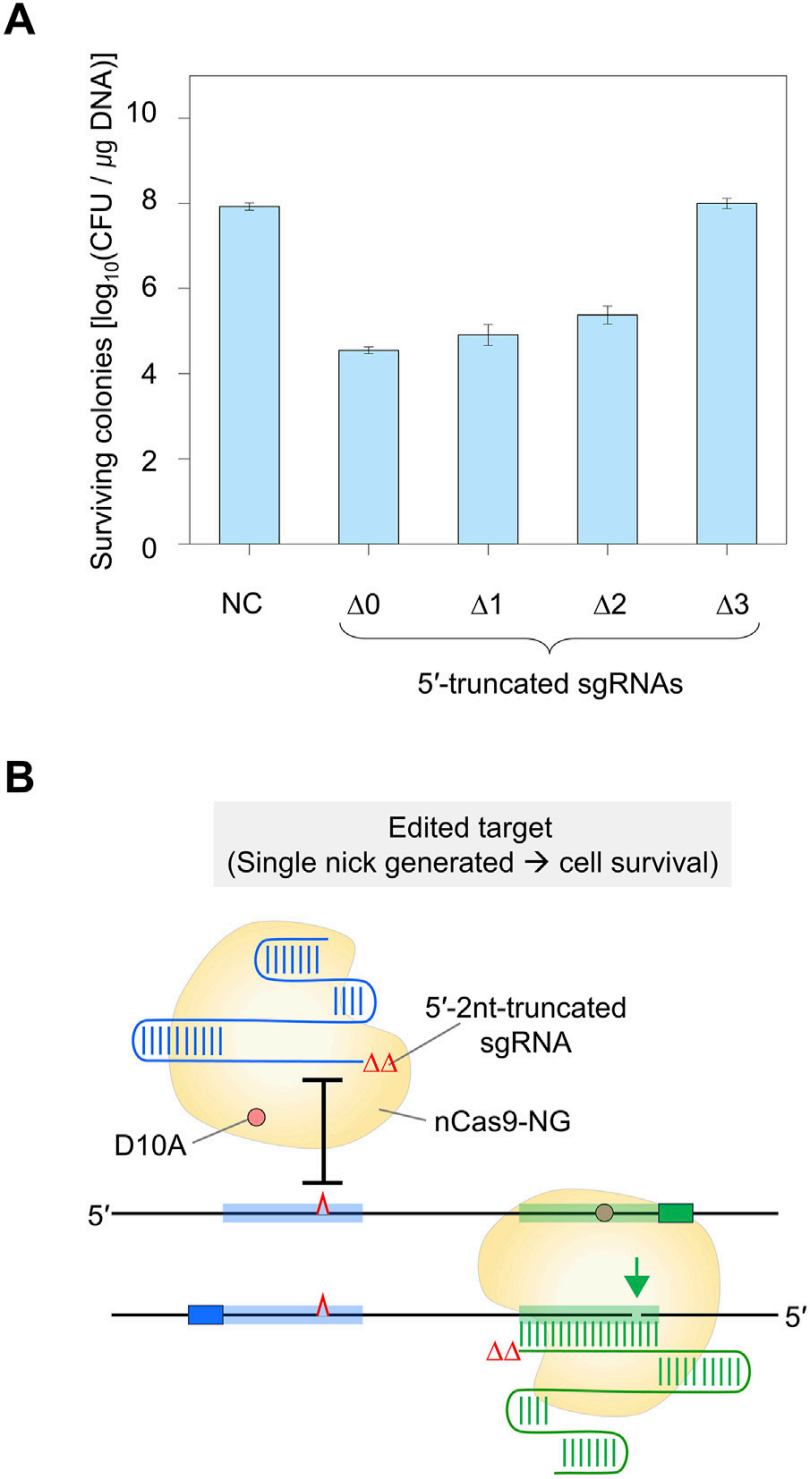
DNA cleavage efficiency of Cas9-NG nickase using 5′-truncated dual sgRNA. **(A)** Determination of minimum TRS length of dual sgRNA required for the formation of double nicks. ∆0 to ∆3 represent the number of truncated nucleotides at the 5′-end of the dual sgRNAs. Negative control (NC) used a dual sgRNA plasmid targeting the *galK* and *xylB* genes. **(B)** Single-mismatch intolerance of Cas9-NG nickase. The maximally 5′-truncated sgRNA (Δ2)/Cas9-NG nickase complex does not generate a nick in the single-nucleotide-edited target (blue-shaded) and generates a nick in the perfect matched target (green-shaded).TRS stands for target recognition sequence.

**FIGURE 5:**
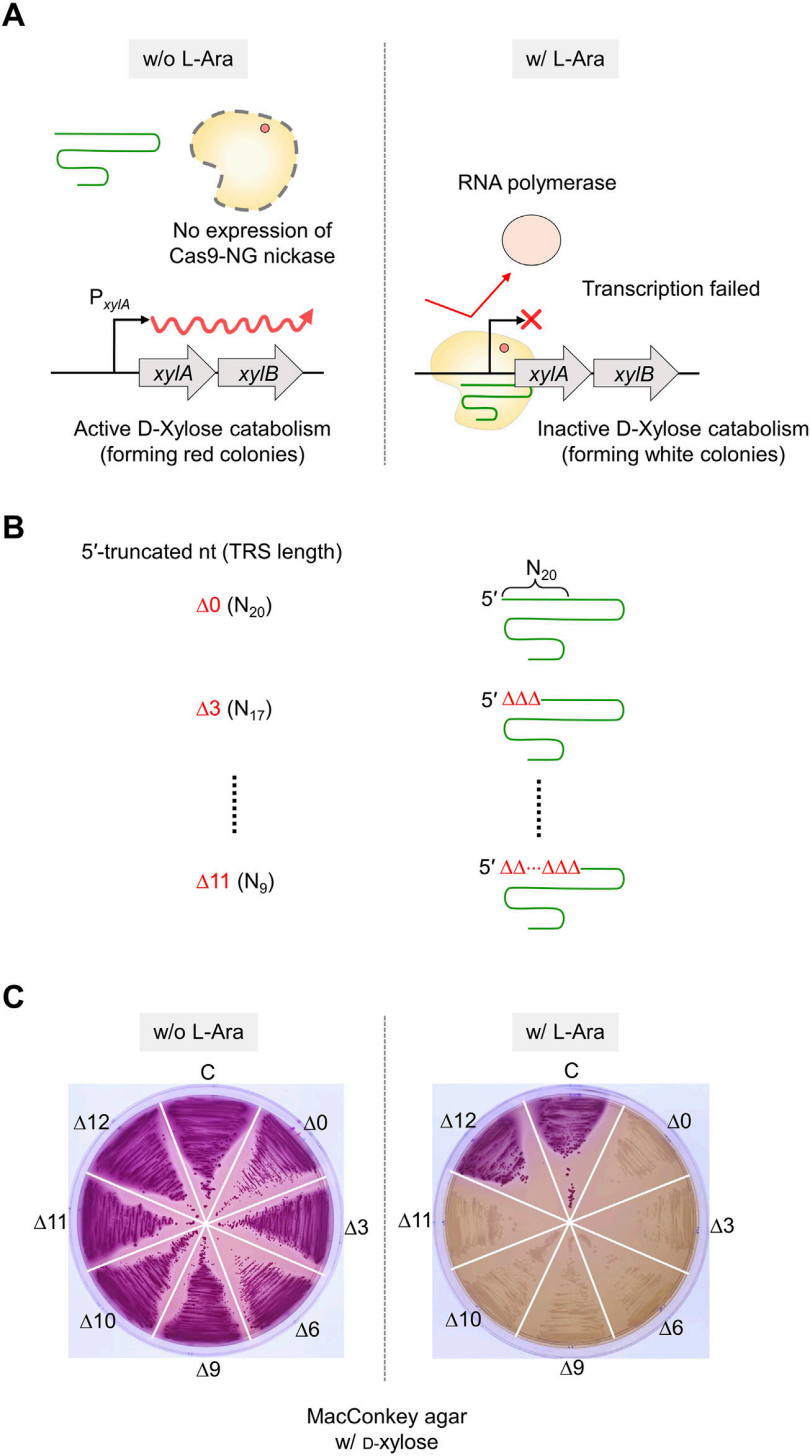
The effect of 5′-truncated sgRNA with different lengths of TRS on target DNA binding activity of sgRNA/Cas9-NG nickase complex. **(A)** Transcriptional repression of xylose operon by sgRNA/Cas9-NG nickase complex. sgRNA/Cas9-NG nickase complex can bind to the *xylA* promoter and repress the transcription of the *xylAB* gene when L-arabinose is present in the medium. **(B)** The 5′-truncated sgRNA with various lengths (N_20_, N_17_, N_14_, N_11_, N_10_, N_9_, and N_8_) of TRS. **(C)** Suppression of *xylAB* gene expression by 5′-truncated sgRNA/Cas9-NG nickase complex confirmed in MacConkey agar plates supplemented with D-xylose. White colonies represent the inability to metabolize xylose due to the repression of *xylAB* gene expression. TRS stands for target recognition sequence.

To enhance the accuracy of single-nucleotide editing, we attempted to determine the length of the TRS in dual sgRNAs required to recognize a target and maintain the nickase activity. Based on previous studies (Lee et al., 2021; Lee et al., 2022), we expected that dual sgRNA with the minimum TRS length could exhibit mismatch intolerance between target DNA and sgRNA, leading to enhanced accuracy in single-nucleotide editing. After introducing dual sgRNAs, with truncations of 1–3 nt at the 5′-end, into Cas9-NG(D10A) nickase cells, we assessed the nickase activity in relation to the varied lengths of the 5′-end truncations in the sgRNAs. When dual sgRNAs with a 1 to 2 nt truncation at the 5′-end were transformed, the number of surviving colonies was approximately equal to the number of transformants containing untruncated dual sgRNA (10^4^ CFU/μg DNA). However, the CFU was increased to 10^7^/μg DNA when dual sgRNA with a 3 nt truncation was used (Figure 4A). These data indicate that the nickase activity of the sgRNA/Cas9-NG(D10A) nickase complex can be retained even when the 5′-end of the dual sgRNA is truncated by up to 2 nt. This implies that the truncation of 2 nt at the 5′-end of the dual sgRNA is necessary to enhance the accuracy of single-nucleotide editing (Figure 4B). Additionally, we determined the minimum number of nucleotides required for sgRNA binding to the target using the CRISPR interference system targeting the *xylA* promoter. Consistent with a previous study (Jeong et al., 2023a), we confirmed that gene expression can be repressed, even if the length of the TRS in sgRNA is shortened to 9 nt (Figures 5A–C). These results showed that *in vivo*, the 5′-end-truncated sgRNA/Cas9-NG(D10A) nickase complex can bind to a relatively short target sequence (∼9 bp), but the formation of nicks requires a minimum target length of 18 bp.

### 3.6 Precise genome editing utilizing 5′-truncated sgRNA/Cas9-NG nickase

**FIGURE 6:**
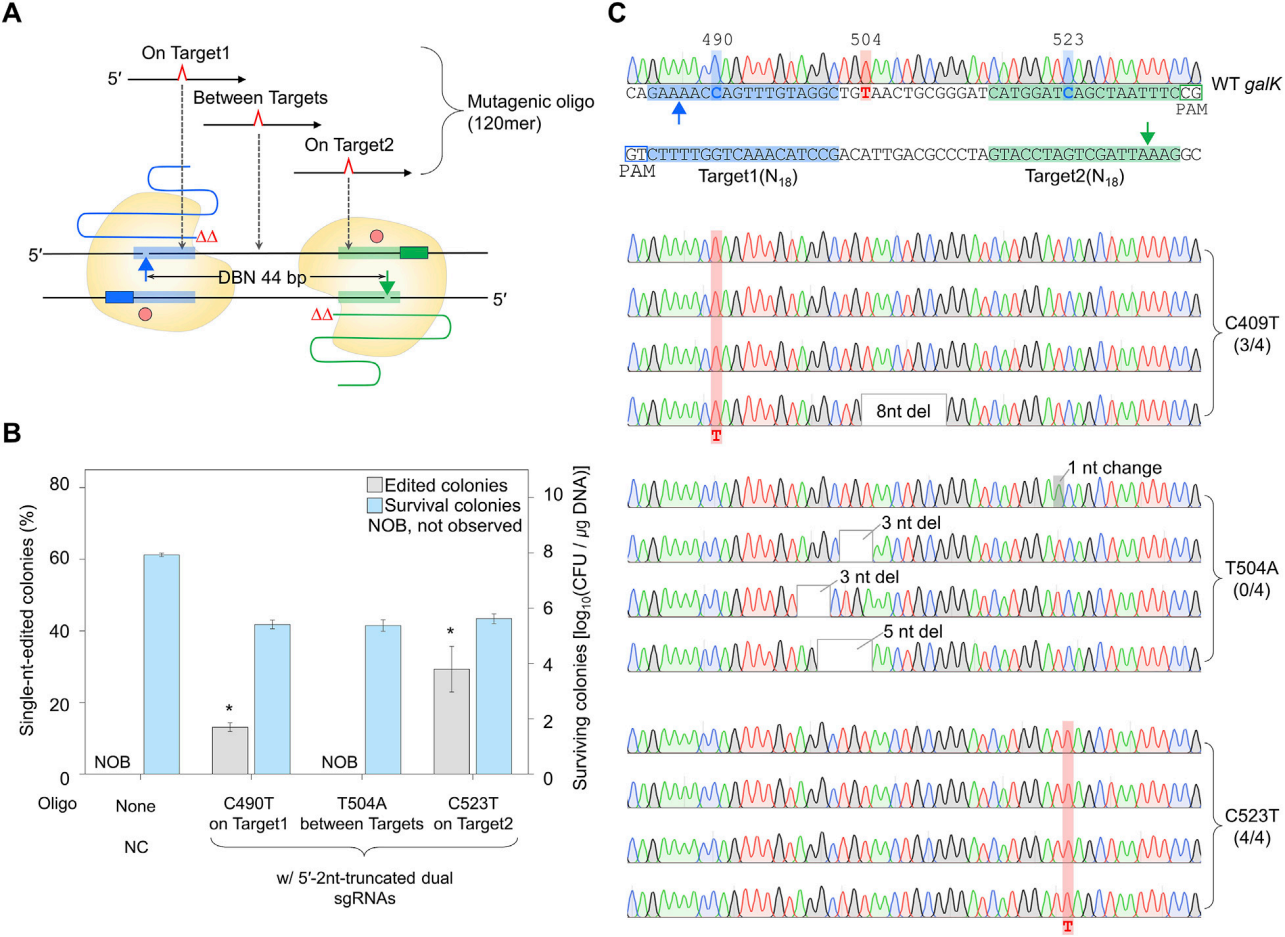
Single-nucleotide editing using the maximally 5′-end-truncated dual sgRNA. **(A)** Schematic representation of single-nucleotide editing at various positions in *galK* target using the maximally truncated dual sgRNA/Cas9-NG nickase complex. Filled triangles indicate the nick sites formed by the maximally truncated sgRNA/Cas9-NG nickase complex. **(B)** The ratio of phenotypic changes and number of surviving colonies caused by single-nucleotide editing in the *galK* gene. Editing accuracy was calculated as the number of colonies with correct editing among randomly selected white colonies confirmed by Sanger sequencing. *P*-values were determined by comparing the ratio of single-nt-edited colonies in on Target1 and on Target2 with those in between Targets. *P < 0.05. Negative control (NC) used a dual sgRNA plasmid targeting the *galK* and *xylB* genes. **(C)** Sanger sequences of single-nucleotide-edited *galK* targets in cells showing white colonies on MacConkey agar containing D-galactose. Bold letters indicate the target nucleotide to be edited. Blue- and green-shaded sequences indicate the target DNA sequences recognized by each sgRNA. Red-shaded chromatograms are correctly edited bases, and altered bases are marked with bold red letters. Gray boxes and gray-shaded chromatograms indicate unwanted mutations. Each bar represents the mean from three independent experiments.

To confirm whether a single nucleotide can be efficiently edited using dual sgRNA with a truncation of 2 nt at the 5′-end, the dual truncated sgRNA plasmid was electroporated with mutagenic oligonucleotides introducing single-nucleotide substitutions at the three mutagenesis sites (Figure 6A). The percentages of single-nt-edited colonies obtained using oligonucleotides that induce mutations in On Target1 and On Target2 with the dual sgRNA truncated by 2 nt at the 5′-end were 13% and 29%, respectively. However, no single-nt-edited colonies were obtained in Between Targets (Figure 6B). The nucleotide sequences of the edited *galK* targets were analyzed using Sanger sequencing. In the case of On Target1 and On Target2, three and four of the four white colonies were correctly edited, respectively. In contrast, in the case of Between Targets, cells from four among four white colonies had unwanted mutations close to the targets (Figure 6C). These results indicated that nickase-mediated single-nucleotide editing could be performed using maximally 5′-end-truncated dual sgRNA and oligonucleotide introducing mutations on the target.

Additionally, genome editing was performed in cells overexpressing Cas9-NG nickase and λ Red using PCR products (∼1 kb) and 5′-end-truncated dual sgRNA plasmid (Supplementary Figure S11A). In the case of On Target1 and On Target2, the percentage of single-nt-edited colonies was 20% and 30%, respectively. In the case of Between Targets, no single-nt edited colonies were obtained on the MacConkey agar containing D-galactose (Supplementary Figure S11B). The accuracy of single-nucleotide editing was confirmed using Sanger sequencing (Supplementary Figure S11C). When the nucleotide to be edited is located on the target, four and four of four white colonies showed correct single-nucleotide substitutions (C490T and C523T), respectively. Conversely, when the nucleotide to be edited was Between Targets, the editing of a single nucleotide was not successful. These data show that Cas9-NG nickase-mediated single-nucleotide editing can be efficiently performed using dual sgRNA truncated by 2 nt at the 5′-end and donor DNA (oligonucleotides or PCR products) containing the nucleotide to be edited on the target sequence.

In addition, we calculated the proportion of single-nucleotide-edited colonies among 10 randomly selected colonies in LB agar. After electroporation of mutagenic oligonucleotide inducing a mutation in Target1 and 5′-truncated sgRNA into pHK463/SH169 cells, we randomly selected 10 candidate colonies that underwent single-nucleotide editing from LB agar containing spectinomycin (Supplementary Figure S12A). Sanger sequencing confirmed that three out of ten randomly selected colonies were accurately edited at a single-nucleotide level (Supplementary Figure S12B), consistent with the editing efficiency determined through phenotypic observation (Figure 6B; Supplementary Figure S11B).

## 4 Discussion

Despite the advantage of reducing off-target effects, precise genome editing using nickase has been rarely studied. We performed systematic experiments in a microbial model to find the optimal positions of nick and target. First, we investigated the relationship between DBN and genome editing efficiency (Figure 1A). When the distance between double nicks was 10–26 bp apart, the proportion of surviving cells was high, and the target editing efficiency calculated by phenotype was low at about 9% (Figures 1B, C). Conversely, a distance of approximately 44–53 bp between two nicks is lethal to unedited cells, enabling the efficient acquisition of edited cells that exhibit a phenotype of white colonies on MacConkey agar (Figures 1B, C). This result was similar to a previous Cas9 nickase study (Schubert et al., 2021), where the editing efficiency was high when sgRNA with a DBN of 40–68 bp apart was used. These data suggest that the formation of double nicks, which can induce cell death, is directly related to the target gene editing.

When the distance between the double nicks decreases, steric hindrance is anticipated to affect the simultaneous recognition and complete cleavage of the target DNA by two distinct Cas9 nickases. Based on the crystal structure of the Cas9-sgRNA-DNA complex previously reported (Nishimasu et al., 2014), it can be seen that when the DBN is 26 bp, the different Cas9 proteins can overlap with each other as they bind to their respective targets. Due to this mutual interference, it can be assumed that DSBs were not generated, thereby increasing the proportion of survival cells. Conversely, when the DBN is 44 bp, the sgRNA/Cas9-NG(D10A) nickase complex acts independently without interfering with each other to form DSBs, reducing the number of surviving colonies and effectively inducing genome editing.

Genome editing was confirmed by the restriction enzyme digestion, but Sanger sequencing results showed that unwanted mutations were introduced close to the target nucleotide sequence (Supplementary Figures S4B, S5). While most nickase studies used a simple restriction enzyme or T7 endonuclease 1 (T7E1) assay (Chiang et al., 2016; Ran et al., 2013a), our study demonstrated that Sanger sequencing method is necessary to confirm the accuracy of nickase-mediated genome editing to verify the introduction of desired mutations at the target site.

We examined the effects of the mutation locations in mutagenic oligos on genome editing efficiency. In the case of On Target1, a high proportion of white colonies was observed when the mutagenic oligo with the extended homologous region was beyond the nick site (Figure 2A). In the case of Between Targets, more than 35% of white colonies were obtained when M67-5′E, M67-3′E, M90, and M120 were used, which is thought to be because the four mutagenic oligos have a sufficient length of homology arm on either side of the mutagenesis sites (Figure 2B). These results indicate that editing can be efficiently performed when homologous regions in the donor DNA are equally balanced on either side of the mutagenic sequence.

In the PAM-in design, even if the distance of double nicks was 48 bp apart, no white colonies were observed, and the number of surviving colonies was high at 10^7.5^ CFU/μg DNA (Figures 1B, C). This implies that the PAM orientation is a crucial factor influencing genome editing. This result is consistent with a previous Cas9(D10A) nickase study, which demonstrated that in the case of PAM-in design, the target editing efficiency was low even when the distance between double nicks was 59 or 66 bp apart (Schubert et al., 2021). A previous studies explained that the low editing efficiency in the PAM-in design was due to the specific overhang type of the target DNA produced by a pair of Cas9 nickases (Cho et al., 2014; Mali et al., 2013; Shen et al., 2014). Given the research findings to date, it is not yet clear whether the observed effects are due to the 5′- and 3′-overhang products generated after the nickase reaction or to differences in the nickase’s directionality when recognizing the target. Therefore, further investigation into this matter is necessary.

In this study, when PAM-out (46) was used, the proportion of white colonies increased dramatically to 40–94% compared to PAM-in (48), and the number of surviving colonies also significantly decreased to 10^5^ CFU/μg DNA (Figures 3A, B). These results align with those obtained using DBN 44 with a PAM-out design, which showed a high percentage of white colonies indicative of edited targets and a decrease in the number of surviving colonies (Figures 1B, C). According to a previous study, HDR-mediated genome editing is efficiently induced when double nicks are simultaneously generated by paired sgRNA in the nickase system (Ran et al., 2013a). Therefore, it can be inferred that with the PAM-out design, two different Cas9 nickases simultaneously form nicks to induce DSB, resulting in a decrease in the number of survival cells and genome editing with high efficiency. In contrast, because cells did not die when PAM-in construction was used, it is assumed that double nicks were not generated simultaneously and, therefore, no mutations were formed at the target site.

When a single nucleotide was edited using N_20_ dual sgRNA, white colonies were observed at a high percentage of over 70% on MacConkey agar supplemented with D-galactose. However, several mutations, including substitutions and deletions, were introduced around the single-nucleotide editing target. In particular, large deletions were mostly generated in the target sequence recognized by sgRNA (Supplementary Figure S10A, B). The CRISPR-Cas system possesses mismatch tolerance, meaning it can recognize and cleave the target even with 1-2 mismatches between the target DNA and gRNA (Fu et al., 2013; Lee et al., 2020). It has been reported that when there are more than four mismatches between the target and the gRNA, the activation of Cas9 nuclease is inhibited, allowing the acquisition of the edited target without cleavage (Ricci et al., 2019). However, since a single base mismatch can still be cleaved by Cas9 nickase, it is expected that a single base edited target would be cleaved and thus unable to introduce the mutation. Cho et al. (2014) reported that double nicks by Cas9 nickase cause small deletions around the target sequence, and the target site remains intact during this process, so nickase can repeatedly induce DSBs until the target sites are deleted. Similar to previous study, the observed mutations in our target sequences can be attributed to the dual sgRNA/Cas9-NG(D10A) nickase complex continually cleaving the target site. This process, coupled with the indel mutations arising during the repair process, may result in large deletion mutations until the sgRNA can no longer recognize the target nucleotide sequence.

Based on previous our study (Lee et al., 2021), we anticipated that the maximally truncated dual sgRNA/nickase complex might be effective for the negative selection of single-nucleotide-edited cells. We attempted single-nucleotide editing at various positions in the *galK* target (Figure 6A; Supplementary Figure S11A). When donor DNAs that cause mutations on the target sequence where the sgRNA hybridizes were used, only the target nucleotide was precisely edited (Figure 6C; Supplementary Figure S11C). In the case of mutations introduced between targets outside the target sequence, it is thought that the truncated sgRNA/Cas9 nickase complex continuously cleaves the target DNA, potentially leading to indel formation during the repair process. However, when mutations are introduced on target, the binding and cleavage efficiency of the truncated sgRNA/Cas9 nickase is reduced, preventing further cleavage of the target DNA, thereby allowing the effective acquisition of single-nucleotide-edited cells (Figure 4B).

In addition, upon examining the target sequence of colonies randomly selected from LB medium, we confirmed successful editing at the single-nucleotide level using maximally truncated dual sgRNA (Supplementary Figure S12B). When untruncated sgRNA was used, unwanted mutations occurred in the DNA sequence where the sgRNA binds, separate from the editing of the target nucleotide (Supplementary Figure S10B). However, when the maximally truncated dual sgRNA was used, it was confirmed that in all seven unedited colonies, only the target nucleotide remained unedited without any unwanted mutations (Supplementary Figure S12B). Based on these results, we inferred that the *in vivo* activity of maximally truncated sgRNA/nickase is relatively weaker than that of untruncated sgRNA/nickase, enabling donor DNA-dependent accurate genome editing without unwanted mutations in the surrounding sequences.

Additionally, using chemically modified oligonucleotides or DNA, such as base methylation and 5′ or 3′-modifications, could enhance editing efficiency by preventing cleavage by nucleases and improving *in vivo* stability, as the donor DNA is recognized as the parental strand during the DNA repair process. Further research utilizing chemically modified donor DNA will be necessary for precise and efficient genome editing.

Our study provides important insights into future research directions for the development and optimization of various genome editing technologies based on nickase, such as base editors (BE) click editor (CE), and prime editors (PE). BE, CE, and PE may induce off-target mutations due to the mismatch tolerance inherent in the CRISPR-Cas system. This issue is particularly significant in eukaryotes with large genomes, as these organisms often contain sequences similar to the target sequence, thereby increasing the likelihood of off-target editing (Fu et al., 2013; Zhu et al., 2024). Utilizing truncated sgRNA can help reduce off-target effects and achieve precise editing by minimizing unnecessary nicks and ensuring that single nicks occur only at the desired location.

In addition, an example of BE, known as Td-CBE, consists of nSpCas9 (∼160 kDa), TadA-8e (∼18 kDa), and 2XUGI (∼19 kDa) (Chen et al., 2023). PE5 is composed of nSpCas9 (∼160 kDa), M-MLV reverse transcriptase (71 kDa), pegRNA (∼150 nt), nicking sgRNA (∼100 nt), and MLH1dn (754 aa), a dominant negative MMR protein (Chen et al., 2021). The substantial size of theses constructs presents challenges for their incorporation into vectors, such as adeno-associated virus (AAV) vectors, lipid nanoparticles (LNPs), enveloped delivery vehicles (EDVs) (Tsuchida et al., 2024) and for their delivery into cells. The paired Cas9-NG nickase and truncated sgRNA system may offer a possible approach to address some of the issues associated with BE and PE because it requires only SpCas9-NG nickase moiety (∼160 kDa) and smaller truncated sgRNA (∼100 nt) for genome editing.

We demonstrated that single-nucleotide editing can be successfully performed with effective negative selection by 1) designing the desired target using Cas9-NG nickase, 2) forming *in vivo* double nicks through PAM-out design, and 3) using truncated sgRNA to overcome the mismatch tolerance problem. The target sequence length of 36 nt for truncated sgRNA and paired nickase is still sufficient to identify unique targets within the genomes of various organisms. Therefore, our study demonstrates that nickase, which can reduce off-target editing, can be efficiently used to edit any sequence in the genome to a desired sequence at the single-nucleotide level. This study will especially serve as a crucial foundation for efforts to enhance the efficiency and accuracy of single-nucleotide genome editing.

## Data Availability

The original contributions presented in the study are included in the article/Supplementary Material, further inquiries can be directed to the corresponding author.
