# Supplementary material for: Use of paired Cas9-NG nickase and truncated sgRNAs for single-nucleotide microbial genome editing
Source: Front Genome Ed. 2024 Sep 26;6:1471720. doi: 10.3389/fgeed.2024.1471720 (PMC11464485; doi:10.3389/fgeed.2024.1471720)
Supplement: Supplementary file 1 [file DataSheet1.pdf]

## Supplementary Material

# Use of paired Cas9-NG nickase and truncated sgRNAs for single-nucleotide microbial genome editing

Song Hee Jeong, Ho Joung Lee, and Sang Jun Lee*

Department of Systems Biotechnology, Institute of Microbiomics,
Chung-Ang University, Anseong 17546, Republic of Korea

*Corresponding author : sangjlee@cau.ac.kr

**Supplementary Table S1.** Strains and plasmids used in this study.

| Name | Characteristics | Source/reference |
|---|---|---|
| Strain | | |
| *Escherichia coli* | | |
| DH5α | *fhuA2 lac(del)U169 phoA glnV44 Φ80' lacZ(del)M15 gyrA96 recA1 relA1 endA1 thi-1 hsdR17* | Laboratory stock |
| MG1655 | F− *ilvG rfb*-50 *rph*-1 | S. Adhya |
| HK1159 | MG1655, *araBAD*::$P_{BAD}$-*cas9-NG*-$\text{Km}^{R}$ | (Lee et al., 2021)[1] |
| SH169 | MG1655, *araBAD*::$P_{BAD}$-*cas9-NG(D10A)*-$\text{Km}^{R}$ | This study |
| Plasmid | | |
| pKD46 | pSC101 *ori* $^{ts}$, *araC*, *λ red genes*, $\text{Amp}^{R}$ | (Datsenko and Wanner, 2000)[2] |
| pHK463 | pSC101 *ori* $^{ts}$, *araC*, *λ bet gene*, $\text{Amp}^{R}$ | (Kim et al., 2020)[3] |
| pHL143 | pSC101 *ori* $^{ts}$, sgRNA target ($N_{20}$, $^{13}$TACTCAATAGGCTTAGATAT$^{32}$ in *cas9-NG*), $\text{Sp}^{R}$ | This study |
| pHL308 | pBR322 *ori*, SpR | (Jeong et al., 2023a)[4] |
| pSH035 | pBR322 *ori*, sgRNA target ($N_{20}$, $^{-66}$AAAGGGAGTGCCCAATATTA$^{-47}$ in $P_{xylA}$), $\text{Sp}^{R}$ | |
| pSH036 | pBR322 *ori*, sgRNA target ($N_{10}$, $^{-56}$CCCAATATTA$^{-47}$ in $P_{xylA}$), $\text{Sp}^{R}$ | |
| pSH037 | pBR322 *ori*, sgRNA target ($N_{9}$, $^{-55}$CCAATATTA$^{-47}$ in $P_{xylA}$), $\text{Sp}^{R}$ | |
| pSH038 | pBR322 *ori*, sgRNA target ($N_{8}$, $^{-54}$CAATATTA$^{-47}$ in $P_{xylA}$), $\text{Sp}^{R}$ | |
| pSH039 | pBR322 *ori*, sgRNA target ($N_{7}$, $^{-53}$AATATTA$^{-47}$ in $P_{xylA}$), $\text{Sp}^{R}$ | |
| pSH086 | pBR322 *ori*, sgRNA target ($N_{17}$, $^{-63}$GGGAGTGCCCAATATTA$^{-47}$ in $P_{xylA}$), $\text{Sp}^{R}$ | |
| pSH087 | pBR322 *ori*, sgRNA target ($N_{14}$, $^{-60}$AGTGCCCAATATTA$^{-47}$ in $P_{xylA}$), $\text{Sp}^{R}$ | |
| pSH088 | pBR322 *ori*, sgRNA target ($N_{11}$, $^{-57}$GCCCAATATTA$^{-47}$ in $P_{xylA}$), $\text{Sp}^{R}$ | |
| pSR017 | pBR322 *ori*, sgRNA targets ($N_{20}$, $^{498}$AGGCTGTAACTGCGGGATCA$^{517}$ in *galK*), $\text{Sp}^{R}$<br>($N_{20}$, $^{638}$TACCTGAAGTTGCGAAAGCG$^{657}$ in *xylB*), $\text{Sp}^{R}$ | (Lim et al., 2023)[5] |
| pSH316 | pBR322 *ori*, sgRNA targets ($N_{20}$, $^{511}$CGCAGTTACAGCCTACAAAC$^{492}$ in *galK*), $\text{Sp}^{R}$<br>($N_{20}$, $^{498}$AGGCTGTAACTGCGGGATCA$^{517}$ in *galK*), $\text{Sp}^{R}$ | This study |
| pSH317 | pBR322 *ori*, sgRNA targets ($N_{20}$, $^{511}$CGCAGTTACAGCCTACAAAC$^{492}$ in *galK*), $\text{Sp}^{R}$<br>($N_{20}$, $^{488}$ACCAGTTTGTAGGCTGTAAC$^{507}$ in *galK*), $\text{Sp}^{R}$ | This study |
| pSH323 | pBR322 *ori*, sgRNA targets ($N_{20}$, $^{511}$CGCAGTTACAGCCTACAAAC$^{492}$ in *galK*), $\text{Sp}^{R}$<br>($N_{20}$, $^{478}$GAAGCAGAAAACCAGTTTGT$^{497}$ in *galK*), $\text{Sp}^{R}$ | This study |
| pSH324 | pBR322 *ori*, sgRNA targets ($N_{20}$, $^{511}$CGCAGTTACAGCCTACAAAC$^{492}$ in *galK*), $\text{Sp}^{R}$<br>($N_{20}$, $^{471}$CGGTCAGGAAGCAGAAAACC$^{490}$ in *galK*), $\text{Sp}^{R}$ | This study |
| pSH325 | pBR322 *ori*, sgRNA targets ($N_{20}$, $^{511}$CGCAGTTACAGCCTACAAAC$^{492}$ in *galK*), $\text{Sp}^{R}$<br>($N_{20}$, $^{463}$GCGCTTAACGGTCAGGAAGC$^{482}$ in *galK*), $\text{Sp}^{R}$ | This study |
| pSH329 | pBR322 *ori*, sgRNA targets ($N_{20}$, $^{511}$CGCAGTTACAGCCTACAAAC$^{492}$ in *galK*), $\text{Sp}^{R}$<br>($N_{20}$, $^{504}$TAACTGCGGGATCATGGATC$^{523}$ in *galK*), $\text{Sp}^{R}$ | This study |
| pSH356 | pBR322 *ori*, sgRNA targets ($N_{20}$, $^{514}$ATCATGGATCAGCTAATTTC$^{533}$ in *galK*), $\text{Sp}^{R}$<br>($N_{20}$, $^{503}$CAGCCTACAAACTGGTTTTC$^{484}$ in *galK*), $\text{Sp}^{R}$ | This study |
| pSH358 | pBR322 *ori*, sgRNA targets ($N_{20}$, $^{463}$GCGCTTAACGGTCAGGAAGC$^{482}$ in *galK*), $\text{Sp}^{R}$<br>($N_{20}$, $^{544}$TGCCGAGCGCGGAAATTAGC$^{525}$ in *galK*), $\text{Sp}^{R}$ | This study |
| pSH359 | pBR322 *ori*, sgRNA targets ($N_{20}$, $^{516}$CATGGATCAGCTAATTTCCG$^{535}$ in *galK*), $\text{Sp}^{R}$<br>($N_{20}$, $^{496}$CAAACTGGTTTTCTGCTTCC$^{477}$ in *galK*), $\text{Sp}^{R}$ | This study |
| pSH402 | pBR322 *ori*, sgRNA targets ($N_{19}$, $^{515}$TCATGGATCAGCTAATTTC$^{533}$ in *galK*), $\text{Sp}^{R}$<br>($N_{19}$, $^{502}$AGCCTACAAACTGGTTTTC$^{484}$ in *galK*), $\text{Sp}^{R}$ | This study |
| pSH403 | pBR322 *ori*, sgRNA targets ($N_{18}$, $^{516}$CATGGATCAGCTAATTTC$^{533}$ in *galK*), $\text{Sp}^{R}$<br>($N_{18}$, $^{501}$GCCTACAAACTGGTTTTC$^{484}$ in *galK*), $\text{Sp}^{R}$ | This study |
| pSH404 | pBR322 *ori*, sgRNA targets ($N_{17}$, $^{517}$ATGGATCAGCTAATTTC$^{533}$ in *galK*), $\text{Sp}^{R}$<br>($N_{17}$, $^{500}$CCTACAAACTGGTTTTC$^{484}$ in *galK*), $\text{Sp}^{R}$ | This study |
| pSH405 | pBR322 *ori*, sgRNA target ($N_{20}$, $^{514}$ATCATGGATCAGCTAATTTC$^{533}$ in *galK*), $\text{Sp}^{R}$ | This study |
| pSH406 | pBR322 *ori*, sgRNA target ($N_{20}$, $^{503}$CAGCCTACAAACTGGTTTTC$^{484}$ in *galK*), $\text{Sp}^{R}$ | This study |
| pSH411 | pBR322 *ori*, sgRNA targets ($N_{20}$, $^{516}$CATGGATCAGCTAATTTCCG$^{535}$ in *galK*), $\text{Sp}^{R}$<br>($N_{20}$, $^{503}$CAGCCTACAAACTGGTTTTC$^{484}$ in *galK*), $\text{Sp}^{R}$ | This study |

**Supplementary Table S2.** Primers used in this study.

| Name | Sequence(5′→3′) | Description |
|---|---|---|
| pCas9D10A_F | TACTCAATAGGCTTAGATATGTTTTAGAGCTAGAAATAGCAAG | sgRNA plasmid for the construction of nCas9-NG(D10A) cells |
| pCas9D10A_R | ATATCTAAGCCTATTGAGTAACTAGTATTATACCTAGGACTG | |
| galK511-492,20_F | CGCAGTTACAGCCTACAAACGTTTTAGAGCTAGAAATAGCAAG | |
| galK511-492,20_R | GTTTGTAGGCTGTAACTGCGACTAGTATTATACCTAGGACTG | |
| galK498-517,20_F | AGGCTGTAACTGCGGGATCAGTTTTAGAGCTAGAAATAGCAAGT | |
| galK498-517,20_R | CTGATCCCGCAGTTACAGCCTACTAGTATTATACCTAGGACT | |
| galK488-507,20_F | ACCAGTTTGTAGGCTGTAACGTTTTAGAGCTAGAAATAGCAAG | |
| galK488-507,20_R | GTTACAGCCTACAAACTGGTACTAGTATTATACCTAGGACTG | |
| galK478-497,20_F | GAAGCAGAAAACCAGTTTGTGTTTTAGAGCTAGAAATAGCAAG | |
| galK478-497,20_R | ACAAACTGGTTTTCTGCTTCACTAGTATTATACCTAGGACTG | |
| galK471-490,20_F | CGGTCAGGAAGCAGAAAACCGTTTTAGAGCTAGAAATAGCAAG | |
| galK471-490,20_R | GGTTTTCTGCTTCCTGACCGACTAGTATTATACCTAGGACTG | |
| galK463-482,20_F | GCGCTTAACGGTCAGGAAGCGTTTTAGAGCTAGAAATAGCAAG | PCR primers containing target recognition sequence ($N_{20}$). The PCR products were subsequently ligated using Gibson Assembly, resulting in the construction of dual sgRNA plasmids. |
| galK463-482,20_R | GCTTCCTGACCGTTAAGCGCACTAGTATTATACCTAGGACTG | |
| galK504-523,20_F | TAACTGCGGGATCATGGATCGTTTTAGAGCTAGAAATAGCAAG | |
| galK504-523,20_R | GATCCATGATCCCGCAGTTAACTAGTATTATACCTAGGACTG | |
| galK514-533,20_F | ATCATGGATCAGCTAATTTCGTTTTAGAGCTAGAAATAGCAAG | |
| galK514-533,20_R | GAAATTAGCTGATCCATGATACTAGTATTATACCTAGGACTG | |
| galK503-484,20_F | CAGCCTACAAACTGGTTTTCGTTTTAGAGCTAGAAATAGCAAG | |
| galK503-484,20_R | GAAAACCAGTTTGTAGGCTGACTAGTATTATACCTAGGACTG | |
| galK544-525,20_F | TGCCGAGCGCGGAAATTAGCGTTTTAGAGCTAGAAATAGCAAG | |
| galK544-525,20_R | GCTAATTTCCGCGCTCGGCAACTAGTATTATACCTAGGACTG | |
| galK516-535,20_F | CATGGATCAGCTAATTTCCGGTTTTAGAGCTAGAAATAGCAAG | |
| galK516-535,20_R | CGGAAATTAGCTGATCCATGACTAGTATTATACCTAGGACTG | |
| galK496-477,20_F | CAAACTGGTTTTCTGCTTCCGTTTTAGAGCTAGAAATAGCAAG | |
| galK496-477,20_R | GGAAGCAGAAAACCAGTTTGACTAGTATTATACCTAGGACTG | |
| galK517-533,17_F | AGTATGGATCAGCTAATTTCGTTTTAGAGCTAGAAATAGCAAG | |
| galK517-533,17_R | GAAATTAGCTGATCCATACTAGTATTATACCTAGGACTG | |
| galK500-484,17_F | AGT CCTACAAACTGGTTTTCGTTTTAGAGCTAGAAATAGCAAG | |
| galK500-484,17_R | GAAAACCAGTTTGTAGG ACTAGTATTATACCTAGGACTG | |
| galK516-533,18_F | GT CATGGATCAGCTAATTTCGTTTTAGAGCTAGAAATAGCAAG | PCR primers containing 5′-truncated target recognition sequence ($N_{17}$-$N_{20}$). The PCR products were subsequently ligated using Gibson Assembly, resulting in the construction of dual sgRNA plasmids. |
| galK516-533,18_R | GAAATTAGCTGATCCATGACTAGTATTATACCTAGGACTG | |
| galK501-484,18_F | GT GCCTACAAACTGGTTTTCGTTTTAGAGCTAGAAATAGCAAG | |
| galK501-484,18_R | GAAAACCAGTTTGTAGGCACTAGTATTATACCTAGGACTG | |
| galK515-533,19_F | T TCATGGATCAGCTAATTTCGTTTTAGAGCTAGAAATAGCAAG | |
| galK515-533,19_R | GAAATTAGCTGATCCATGAACTAGTATTATACCTAGGACTG | |
| galK502-484,19_F | T AGCCTACAAACTGGTTTTCGTTTTAGAGCTAGAAATAGCAAG | |
| galK502-484,19_R | GAAAACCAGTTTGTAGGCTACTAGTATTATACCTAGGACTG | |
| Sm_ATG_Out | GATACTGGGCCGGCAGGCGCTCCATTGCCC | PCR primers for contruction of single sgRNA plasmids |
| Sm_TAA_Out | GCAATGGAGCGCCTGCCGGCCCAGTATCAG | |
| galK_F | CATCAGCGTGACTACCATCCCTGCGTTG | PCR and sequencing primers for the *galK* target |
| galK_R | CCAGCGAGACCTGACCGCAGAACAGGC | |

**Supplementary Table S3.** Mutagenic oligonucleotides used in this study.

| Name | Sequence(5′→3′) | Length(mer) | Description | |
|---|---|---|---|---|
| Cas9D10A | CCGACGCTATTTGTGCCGATCGCTAAGCCTATTGAGTATTTC | 42 | Mutagenic oligo for construction of nCas9-NG(D10A) from Cas9-NG cells | |
| L44 | ▲AACCAGTGATCAGGCTGTAACTGCGGGATCATGGATCAGCTAAT▼ | 44 | Equivalent to the distance between nicks (DBN) of 44 bp | |
| L67-5′E | CGCTTAACGGTCAGGAAGCAGAA▲AACCAGTGATCAGGCTGTAACTGCGGGATCATGGATCAGCTAAT▼ | 67 | L44 plus 5'-extension (23 mer) | |
| L67-3′E | ▲AACCAGTGATCAGGCTGTAACTGCGGGATCATGGATCAGCTAAT▼TTCCGCGCTCGGCAAGAAAGATC | 67 | L44 plus 3'-extension (23 mer) | Quadruple bases editing oligonucleotides with different length for galK $^{494}$TTGT to GATC mutation |
| L90 | CGCTTAACGGTCAGGAAGCAGAA▲AACCAGTGATCAGGCTGTAACTGCGGGATCATGGATCAGCTAAT▼TTCCGCGCTCGGCAAGAAAGATC | 90 | L44 plus 5'-extension (23 mer) and 3'-extension (23mer) | |
| L120 | ACGGCGCACAAATCGCGCTTAACGGTCAGGAAGCAGAA▲AACCAGTGATCAGGCTGTAACTGCGGGATCATGGATCAGCTAAT▼TTCCGCGCTCGGCAAGAAAGATCATGCCTTGCTGATCG | 120 | L44 plus 5'-extension (38 mer) and 3'-extension (38 mer) | |
| M44 | ▲AACCAGTTTGTAGGCTGATCATGCGGGATCATGGATCAGCTAAT▼ | 44 | Equivalent to the DBN of 44 bp | |
| M67-5′E | CGCTTAACGGTCAGGAAGCAGAA▲AACCAGTTTGTAGGCTGATCATGCGGGATCATGGATCAGCTAAT▼ | 67 | M44 plus 5'-extension (23 mer) | |
| M67-3′E | ▲AACCAGTTTGTAGGCTGATCATGCGGGATCATGGATCAGCTAAT▼TTCCGCGCTCGGCAAGAAAGATC | 67 | M44 plus 3'-extension (23 mer) | Quadruple bases editing oligonucleotides with different length for galK $^{504}$TAAC to ATCA mutation |
| M90 | CGCTTAACGGTCAGGAAGCAGAA▲AACCAGTTTGTAGGCTGATCATGCGGGATCATGGATCAGCTAAT▼TTCCGCGCTCGGCAAGAAAGATC | 90 | M44 plus 5'-extension (23 mer) and 3'-extension (23mer) | |
| M120 | ACGGCGCACAAATCGCGCTTAACGGTCAGGAAGCAGAA▲AACCAGTTTGTAGGCTGATCATGCGGGATCATGGATCAGCTAAT▼TTCCGCGCTCGGCAAGAAAGATCATGCCTTGCTGATCG | 120 | M44 plus 5'-extension (38 mer) and 3'-extension (38 mer) | |
| R120 | CGGCGCACAAATCGCGCTTAACGGTCAGGAAGCAGAA▲AACCAGTTTGTAGGCTGTAACTGCGGGATCATGGATCAGCTGTAGT▼CCGCGCTCGGCAAGAAAGATCATGCCTTGCTGATCGA | 120 | DBN of 46 nt plus 5'-extension (37 mer) and 3'-extension (37 mer) | Quadruple bases editing oligonucleotides for *galK* $^{528}$AATT to GTAG mutation |
| galKC490T | ACGGCGCACAAATCGCGCTTAACGGTCAGGAAGCAGAA▲AACTAGTTTGTAGGCTGTAACTGCGGGATCATGGATCAGCTAAT▼TTCCGCGCTCGGCAAGAAAGATCATGCCTTGCTGATCG | 120 | Single nucleotide editing oligonucleotides for *galK* $^{490}$T target | |
| galKT504A | CTGGACGGCGCACAAATCGCGCTTAACGGTCAGGAAGCAGAA▲AACCAGTTTGTAGGCTGAAACTGCGGGATCATGGATCAGCTAAT▼TTCCGCGCTCGGCAAGAAAGATCATGCCTTGCTG | 120 | Single nucleotide editing oligonucleotides for *galK* $^{504}$T target | |
| galKC523T | ACGGCGCACAAATCGCGCTTAACGGTCAGGAAGCAGAA▲AACCAGTTTGTAGGCTGTAACTGCGGGATCATGGATTAGCTAAT▼TTCCGCGCTCGGCAAGAAAGATCATGCCTTGCTGATCG | 120 | Single nucleotide editing oligonucleotides for *galK* $^{523}$T target | |

*Underlined red sequences indicate designed mutations.

Upright and inverted triangles indicate the position of nicks (DBN 44) in forward and reverse strands, respectively.

References


1. Lee, H. J., Kim, H. J., and Lee, S. J. (2021). Mismatch Intolerance of 5′-Truncated sgRNAs in CRISPR/Cas9 Enables Efficient Microbial Single-Base Genome Editing. *Int. J. Mol. Sci.* 22 (12), 6457.

2. Datsenko, K. A. and Wanner, B. L. (2000). One-step inactivation of chromosomal genes in K-12 using PCR products. *Proc. Natl. Acad. Sci. U. S. A.* 97 (12), 6640–6645.

3. Kim, B., Kim, H. J., and Lee, S. J. (2020). Regulation of Microbial Metabolic Rates Using CRISPR Interference With Expanded PAM Sequences. *Front. Microbiol.* 11, 282.

4. Jeong, S. H., Kim, H. J., and Lee, S. J. (2023a). New Target Gene Screening Using Shortened and Random sgRNA Libraries in Microbial CRISPR Interference. *ACS Synth. Biol.* 12 (3), 800–808.

5. Lim, S. R., Lee, H. J., Kim, H. J., and Lee, S. J. (2023). Multiplex Single-Nucleotide Microbial Genome Editing Achieved by CRISPR-Cas9 Using 5′-End-Truncated sgRNAs. *ACS Synth. Biol*. 12 (7), 2203–2207.

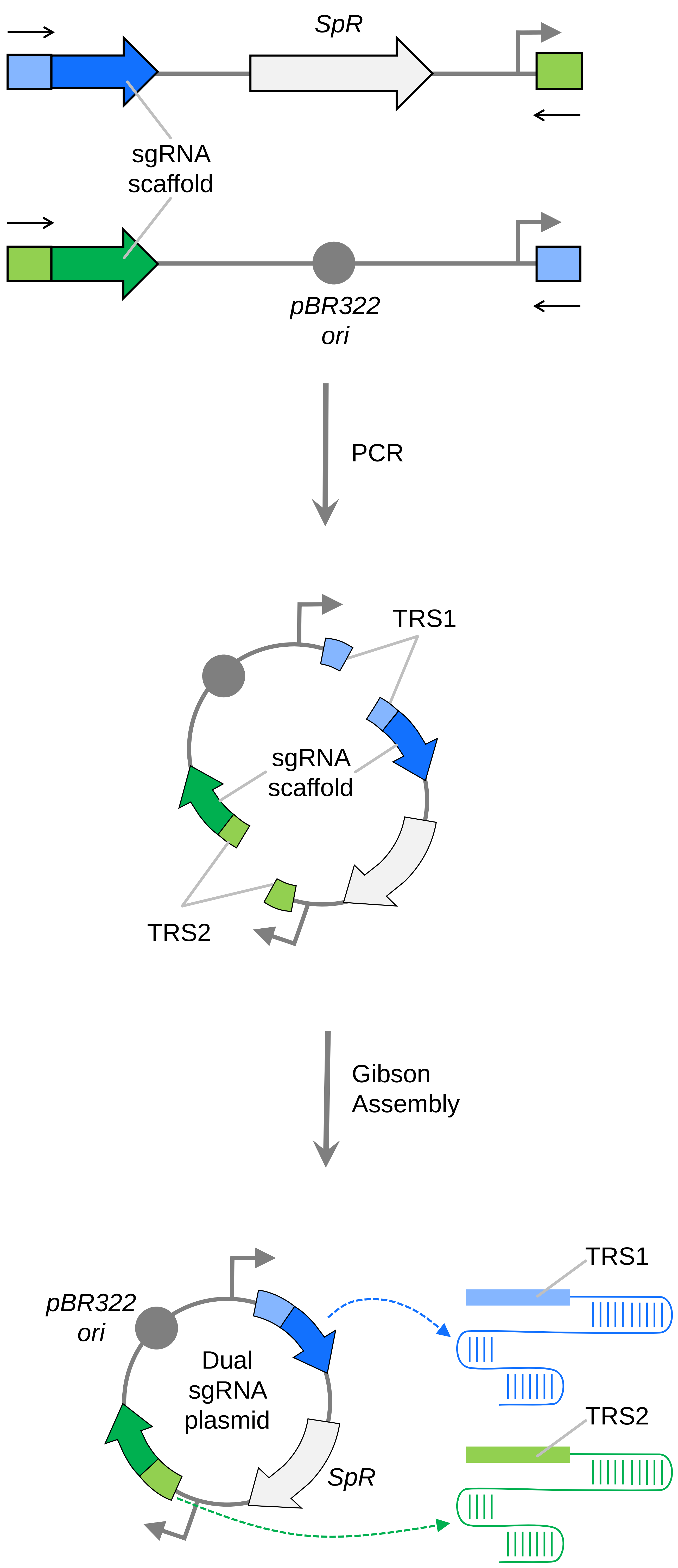


**Supplementary Figure S1.** Construction of dual sgRNA plasmid expressing two sgRNAs that bind to different DNA strands within the same target gene. Dual sgRNA plasmid was constructed by the ligation of two PCR fragments harboring the *ori* or spectinomycin resistance gene. Each end of the fragments is identical to the corresponding $N_{20}$ TRSs. Black arrows indicate the primers used for PCR. TRS stands for target recognition sequence.

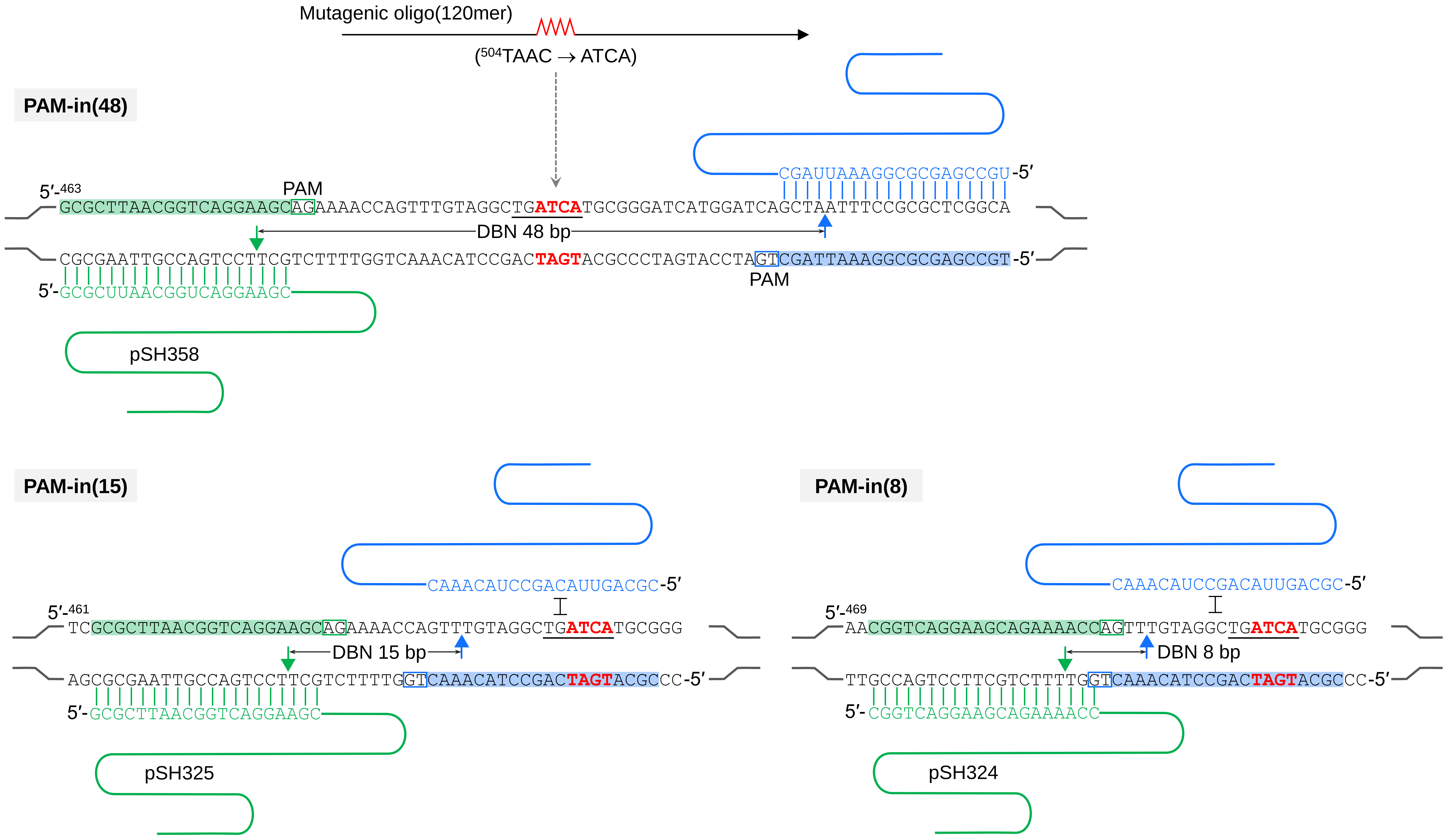


**Supplementary Figure S2.** Various dual sgRNA plasmids with different distances between nicks. Quadruple-base substitutions ($^{504}$TAAC → ATCA) are marked with bold red letters. Underlined sequences indicate the BclI restriction enzyme site. Filled triangles indicate double nicks formed by Cas9-NG nickase and dual sgRNA complex (Continued).

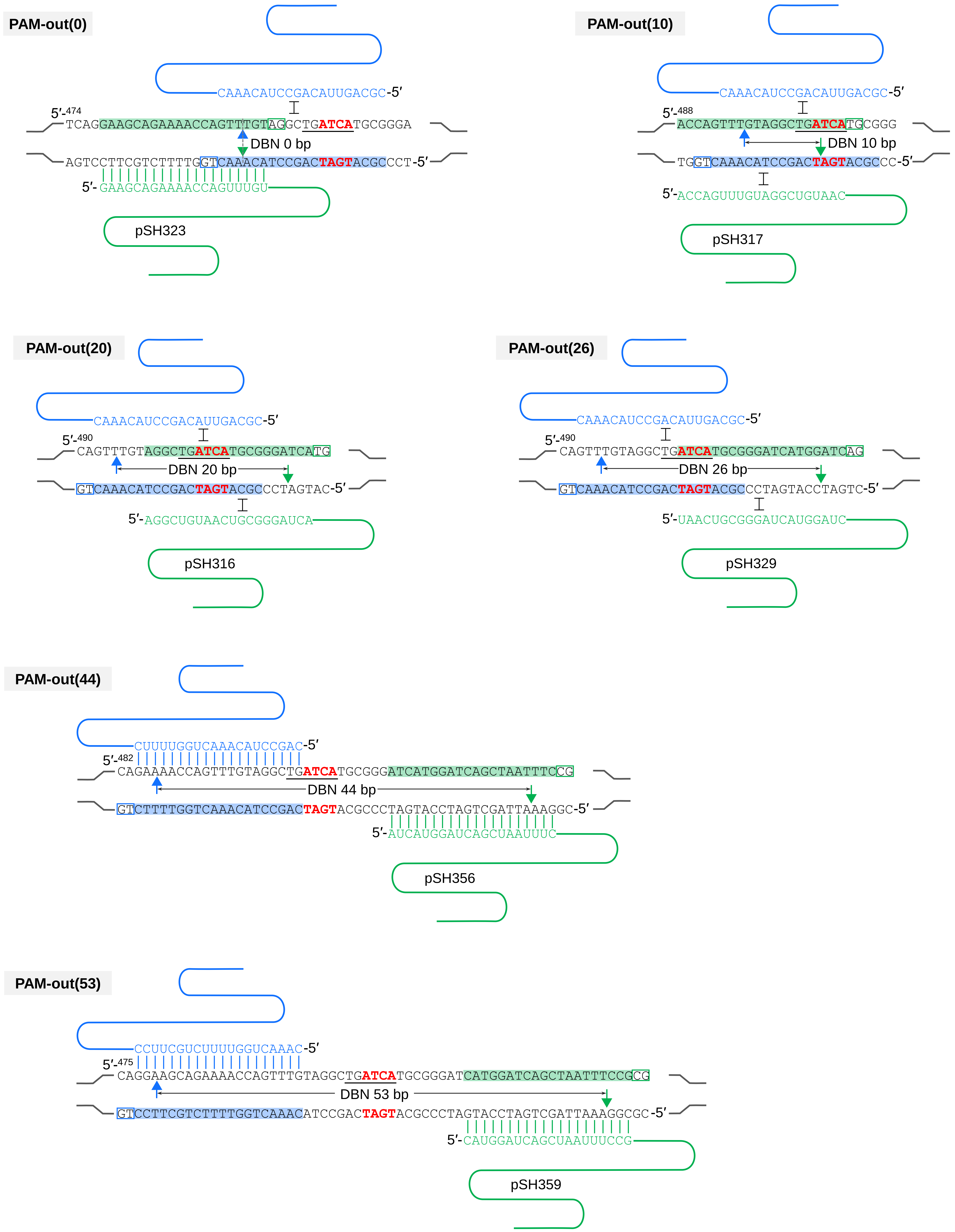


**Supplementary Figure S2.** Various dual sgRNA plasmids with different distances between nicks. Quadruple-base substitutions ([504]TAAC → ATCA) are marked with bold red letters. Underlined sequences indicate the BclI restriction enzyme site. Filled triangles indicate double nicks formed by Cas9-NG nickase and dual sgRNA complex.

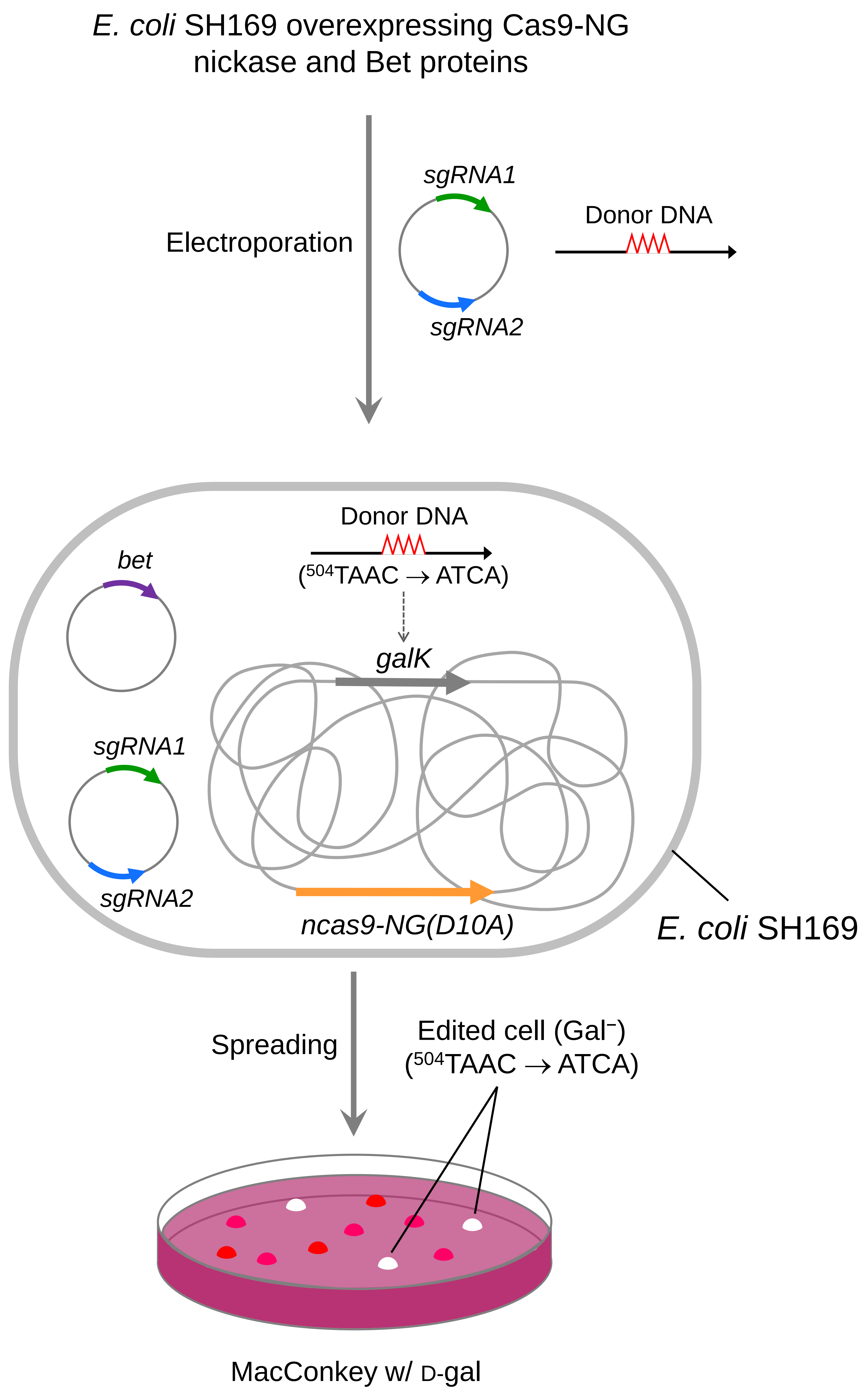


**Supplementary Figure S3.** Genome-editing using dual sgRNA plasmid in Cas9-NG nickase. Dual sgRNA plasmids and mutagenic oligonucleotides carrying quadruple-base mutations ($^{504}$TAAC → ATCA) were electroporated into *Escherichia coli* SH169 cells overexpressing Cas9-NG nickase and Bet proteins. Recovered cells were spread on MacConkey agar plates supplemented with D-galactose. $Gal^+$ and $Gal^-$ cells show red and white colonies on the MacConkey agar, respectively. Editing efficiency was calculated by counting the number of white colonies, which are the phenotypes of cells with successful editing.

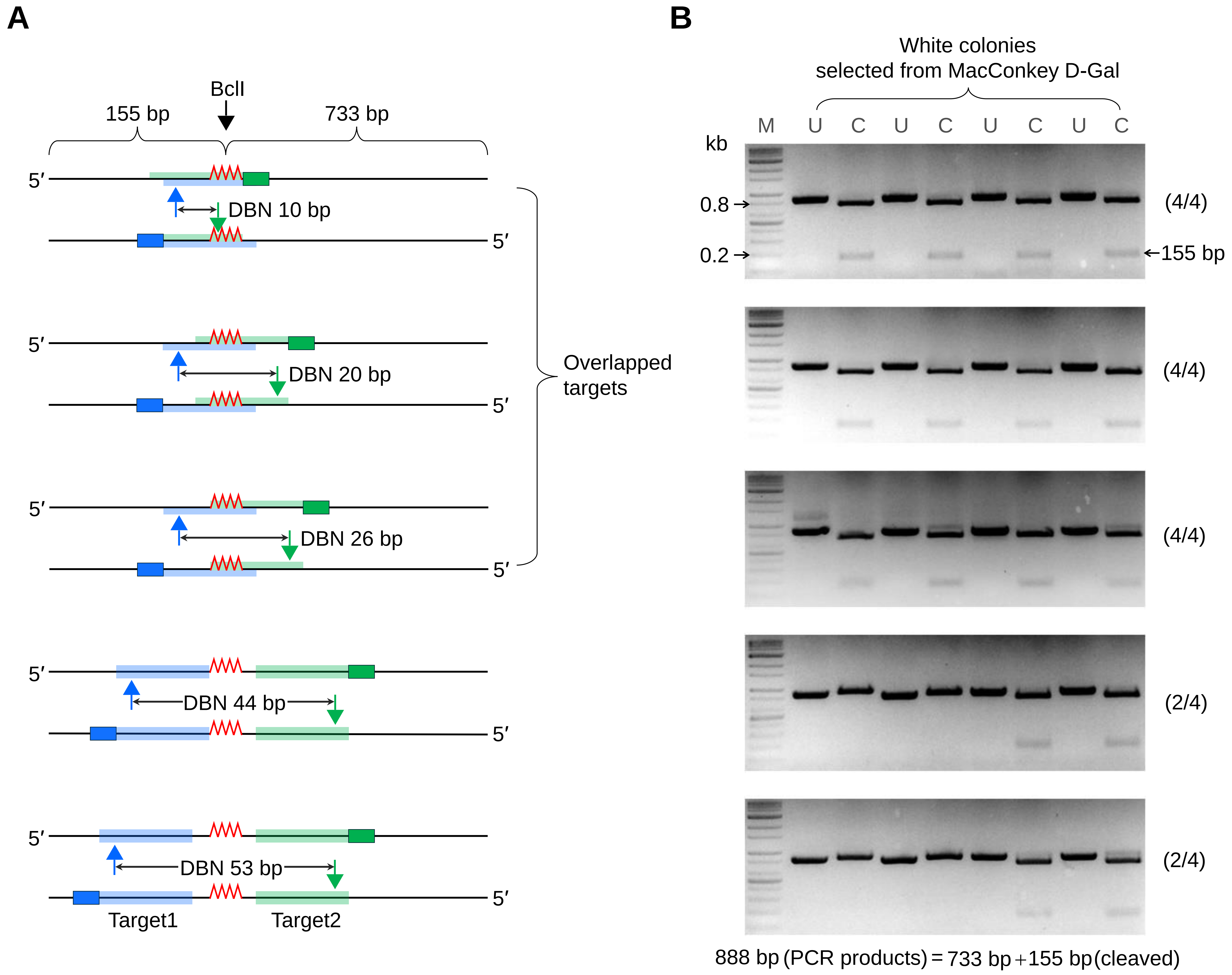


**Supplementary Figure S4.** Confirmation of nickase-mediated genome editing using restriction enzyme digestion. **(A)** Schematic locations of the target DNA sequences recognized by dual sgRNAs with different distances between nicks. Blue- and green-colored boxes indicate the PAM. Filled triangles represent the formation sites of double nicks. Red cones indicate quadruple-base substitutions ($^{504}$TAAC → ATCA) introduced at the target sites. Colony PCR products are cleaved with BclI restriction enzyme. The resulting fragments of edited targets were 155 and 733 bp. **(B)** The 2% agarose gel after digestion of PCR products with BclI restriction enzyme. "U" and "C" indicated uncleaved and cleaved PCR products, respectively. Numbers in parentheses are correctly edited colonies confirmed by restriction enzyme digestion among randomly selected white colonies. PAM stands for protospacer adjacent motif.

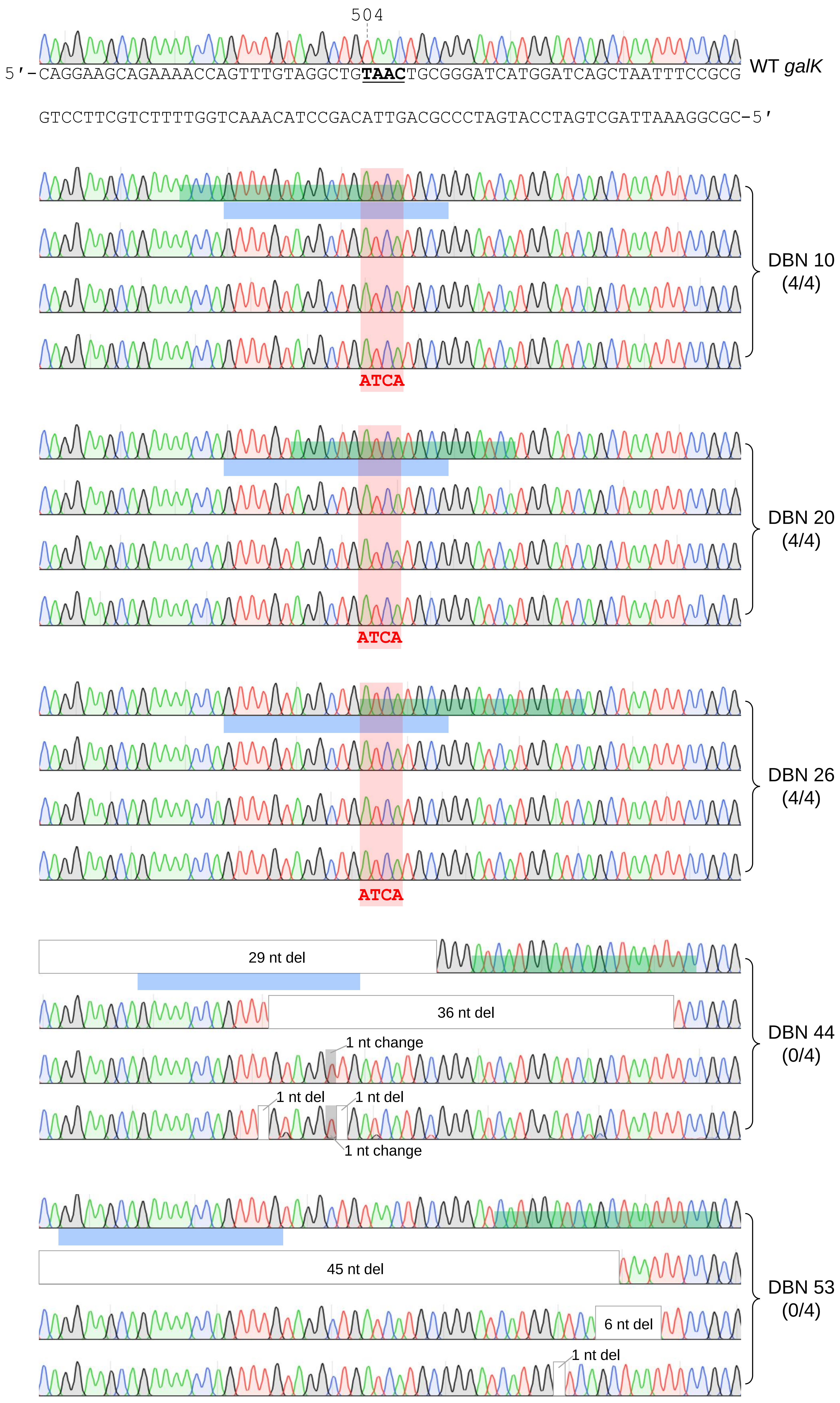


**Supplementary Figure S5.** Sanger sequences of the edited *galK* target with dual sgRNAs carrying different distances between nicks. The underlined bold letters indicate target sequences for genome-editing. Blue- and green-shaded chromatograms indicate the complementary region to the sgRNAs. Red-shaded chromatograms indicate correctly edited bases ($^{504}$TAAC → ATCA). Gray boxes and gray-shaded chromatograms represent unwanted mutations. Numbers in parentheses are correctly edited colonies among white colonies selected for Sanger sequencing. del stands for deletion.

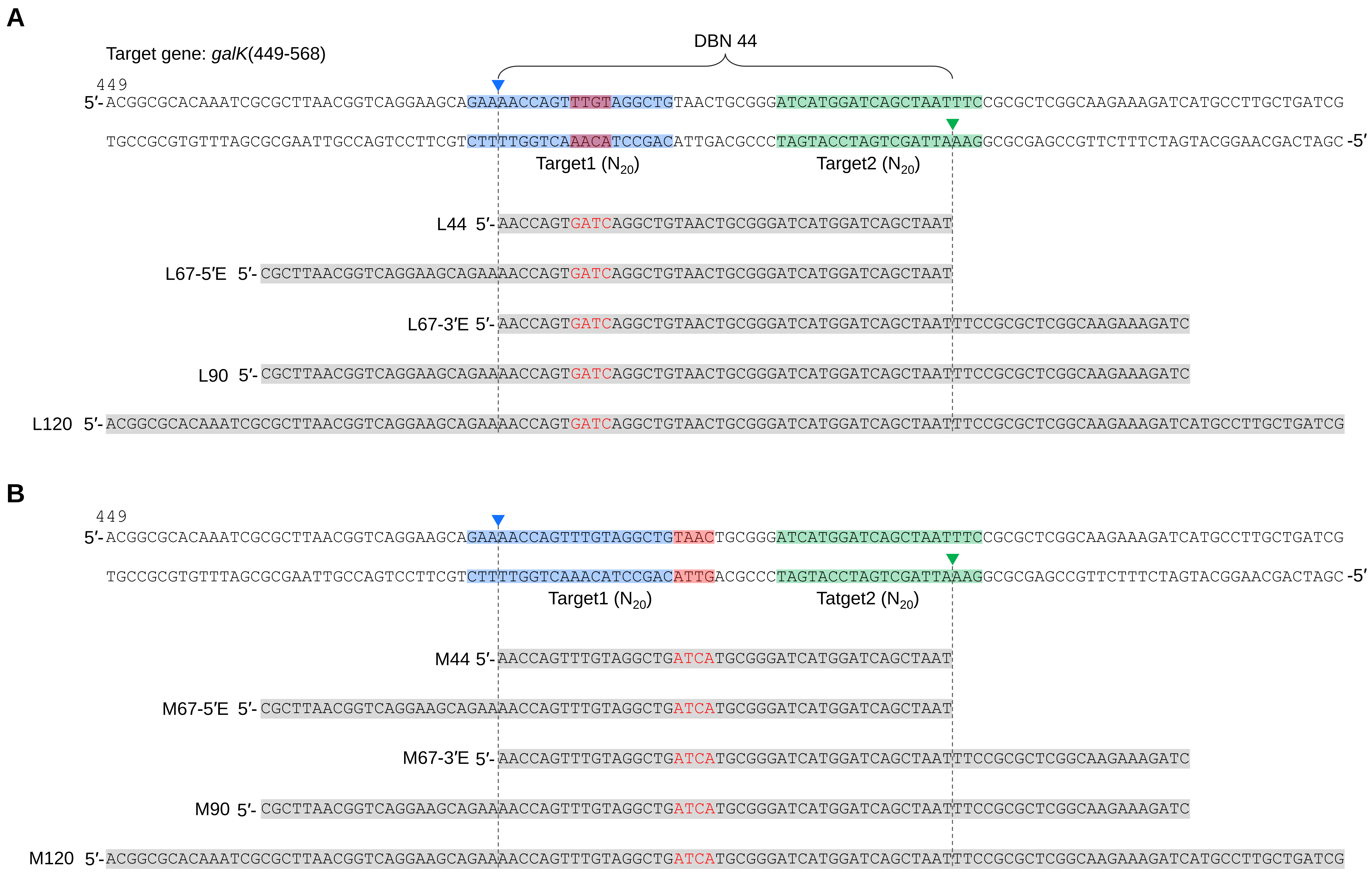


**Supplementary Figure S6.** Mutagenic oligonucleotides with various lengths of homology arms that generate quadruple-base mutations in Target1 **(A)** and Between Targets **(B)**. Blue- and green-shaded letters indicate the complementary region to the sgRNAs. Red-shaded sequences indicate the target nucleotides. Filled triangles represent the formation sites of double nicks. Red letters indicate the designed mutations.

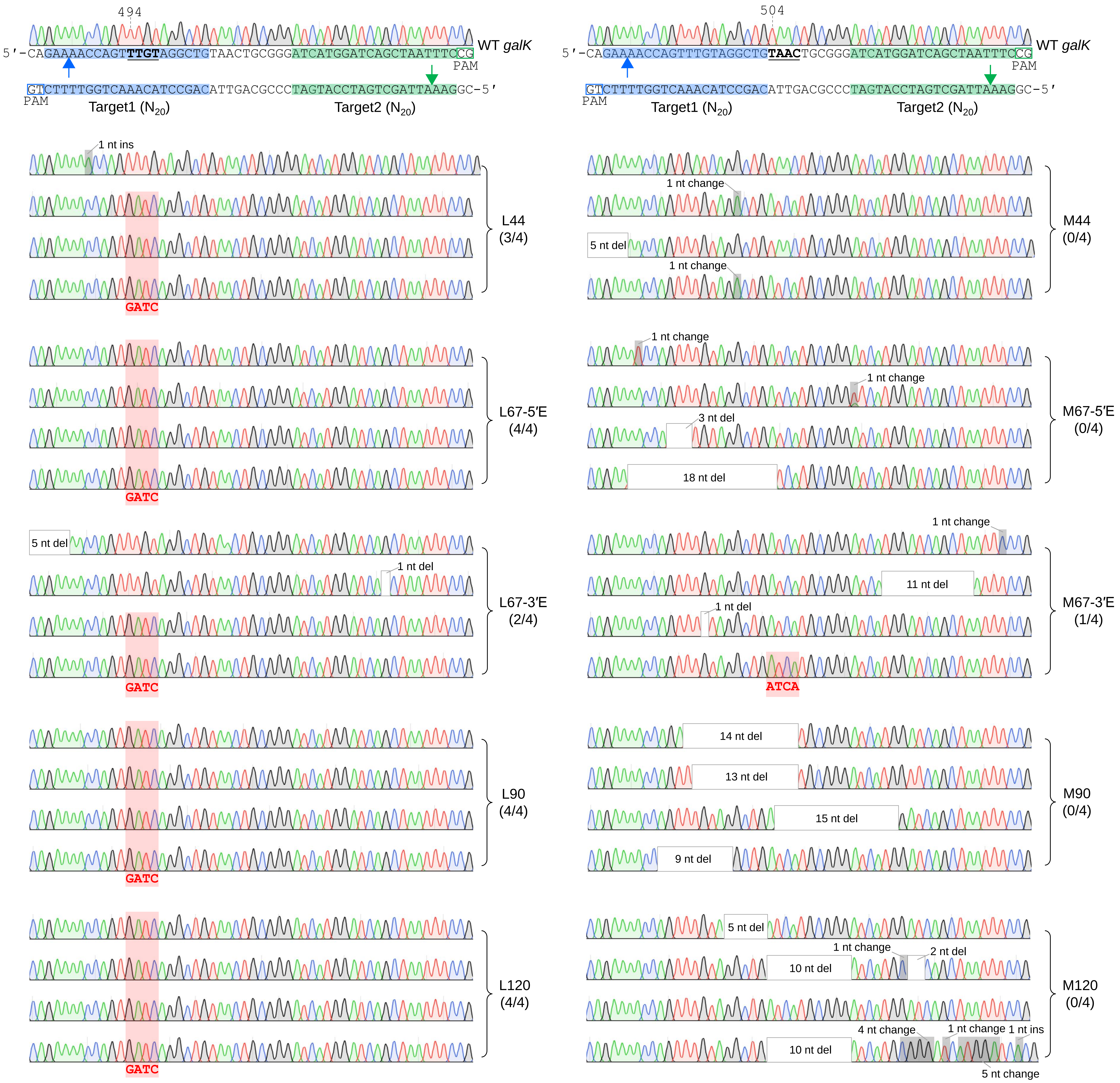


**Supplementary Figure S7.** Sanger sequences of quadruple-base-edited *galK* target using various mutagenic oligonucleotides. Underlined bold letters indicate the target sequences for genome-editing. Blue- and green-shaded sequences indicate the target DNA recognized by each sgRNA. Red-shaded chromatograms represent correctly edited bases. Gray boxes and gray-shaded chromatograms indicate unwanted mutations. Bold red sequences are designed mutations. Numbers in parentheses indicate the number of edited colonies among selected white colonies from MacConkey agar plates supplemented with D-galactose. del and ins stand for deletion and insertion, respectively.

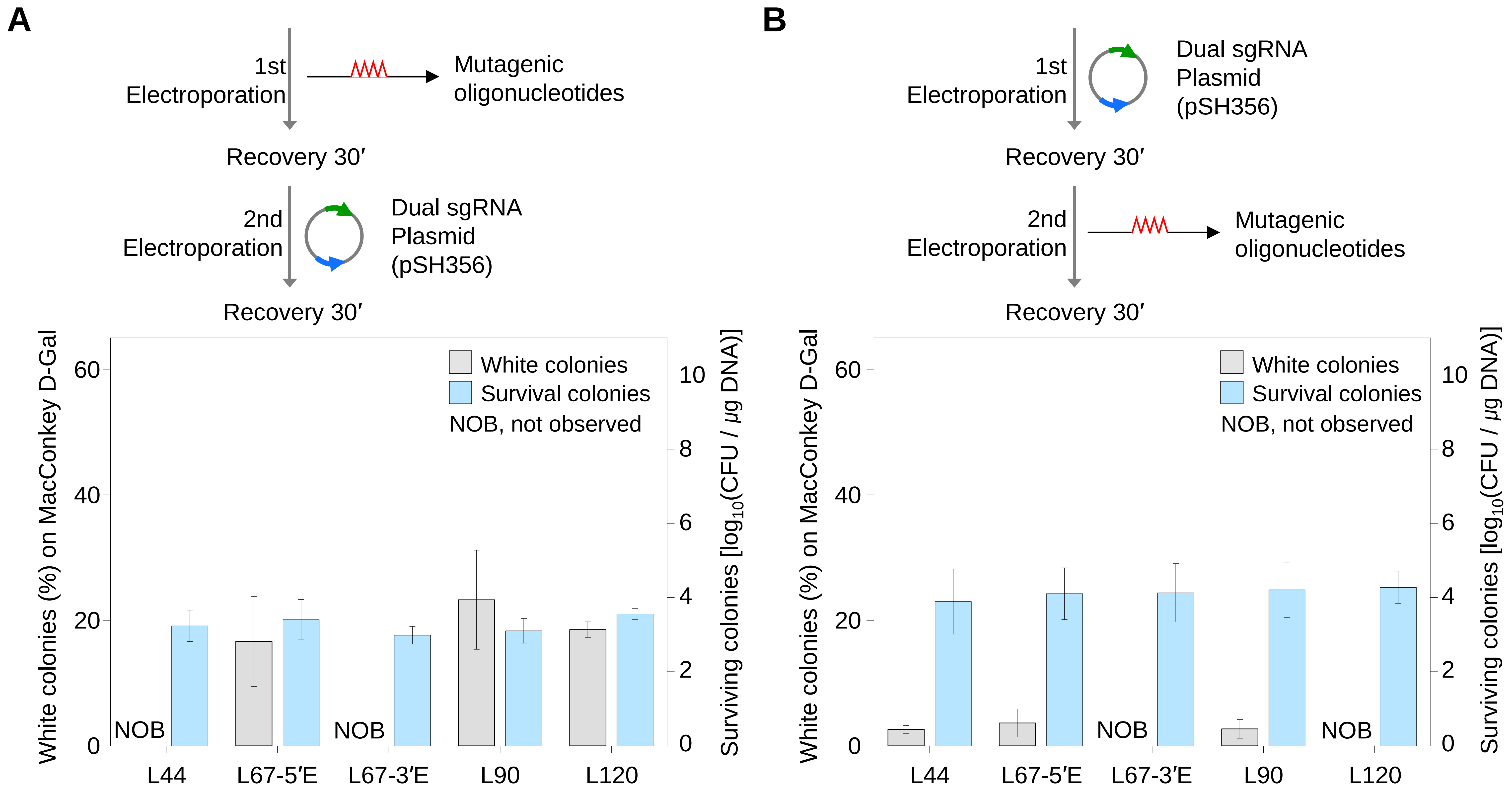


**Supplementary Figure S8.** Comparison of genome editing efficiency depending on the electroporation order of dual sgRNA plasmid and mutagenic oligonucleotides. Genome editing efficiency of the *galK* target was assessed where the mutagenic oligonucleotides are transformed first, followed by the transformation of the dual sgRNA plasmid **(A)**, and where the dual sgRNA plasmid is transformed first, followed by the transformation of mutagenic oligonucleotides later **(B)**. Each bar represents the mean of three independent experiments.

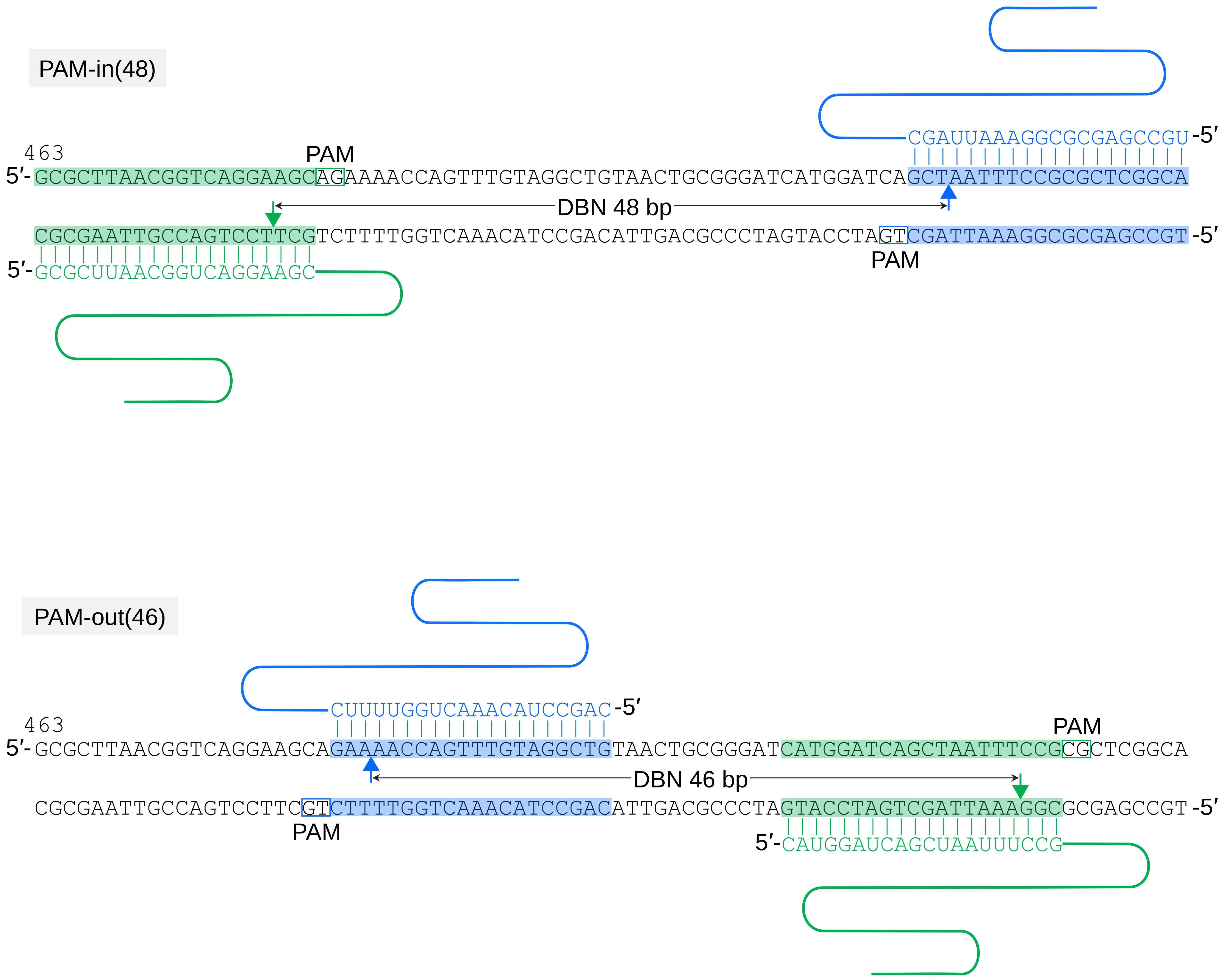


**Supplementary Figure S9.** PAM-in and PAM-out designs with similar distances between nicks. Blue- and green-shaded sequences are the target DNA. Filled triangles indicate the nick sites formed by dual sgRNA/Cas9-NG nickase complex.

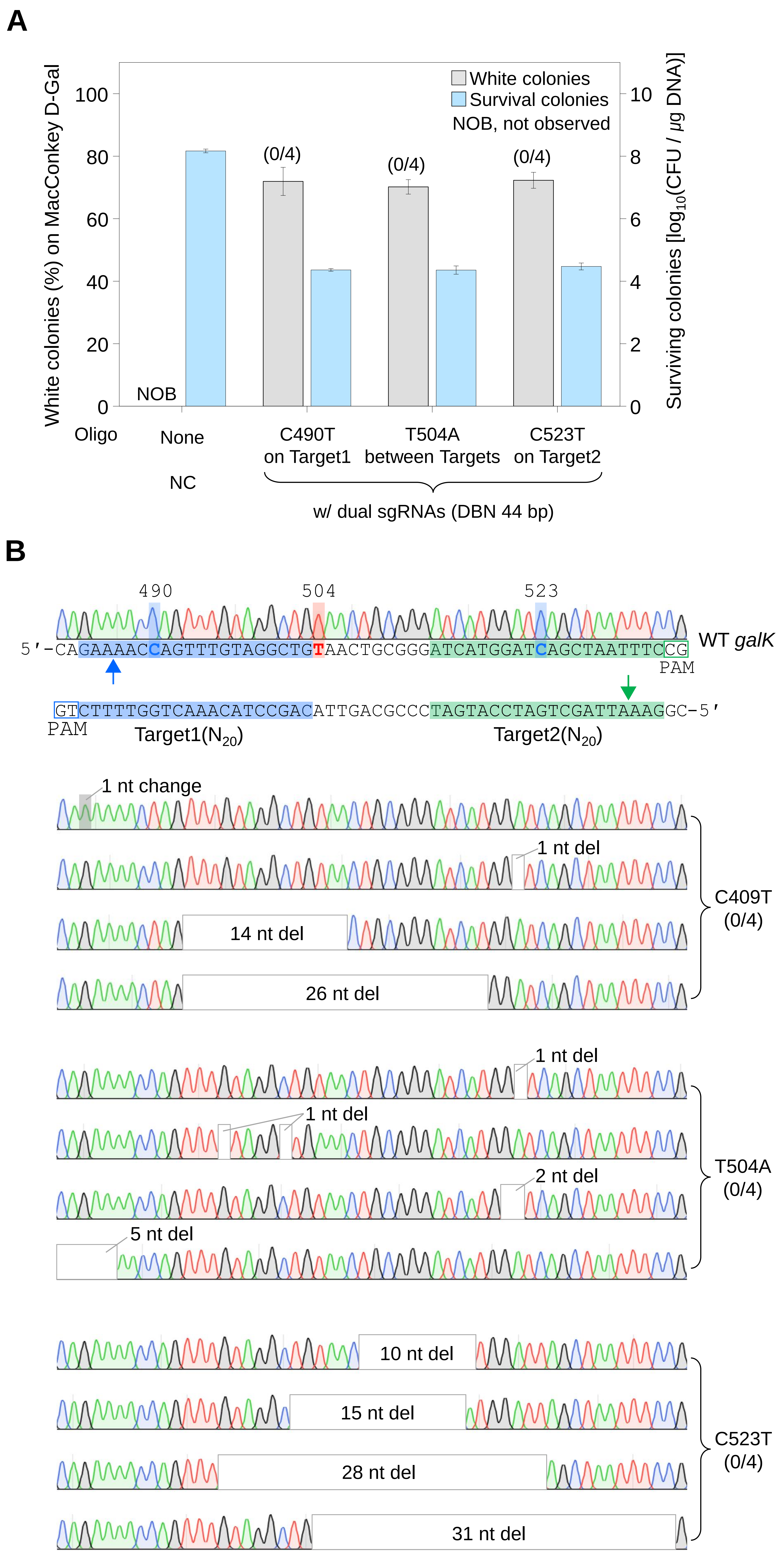


**Supplementary Figure S10.** Attempted single-nucleotide editing by dual sgRNA ($N_{20}$)/Cas9-NG nickase complex. **(A)** Efficiency of Cas9-NG nickase-mediated single-nucleotide editing at various positions in the *galK* target. Editing efficiency was calculated as the proportion of white colonies among total red and white colonies. Numbers in parentheses are correctly edited colonies among white colonies selected for Sanger sequencing. Negative control (NC) used a dual sgRNA plasmid targeting the *galK* and *xylB* genes. **(B)** Sanger sequences of *galK* target in cells showing white colonies on MacConkey agar plates supplemented with D-galactose. Bold sequences indicate the target nucleotides for single-base editing. Blue- and green-shaded sequences indicate the complementary region to the dual sgRNAs. Gray boxes and gray-shaded chromatograms are unwanted mutations. del stands for deletion.

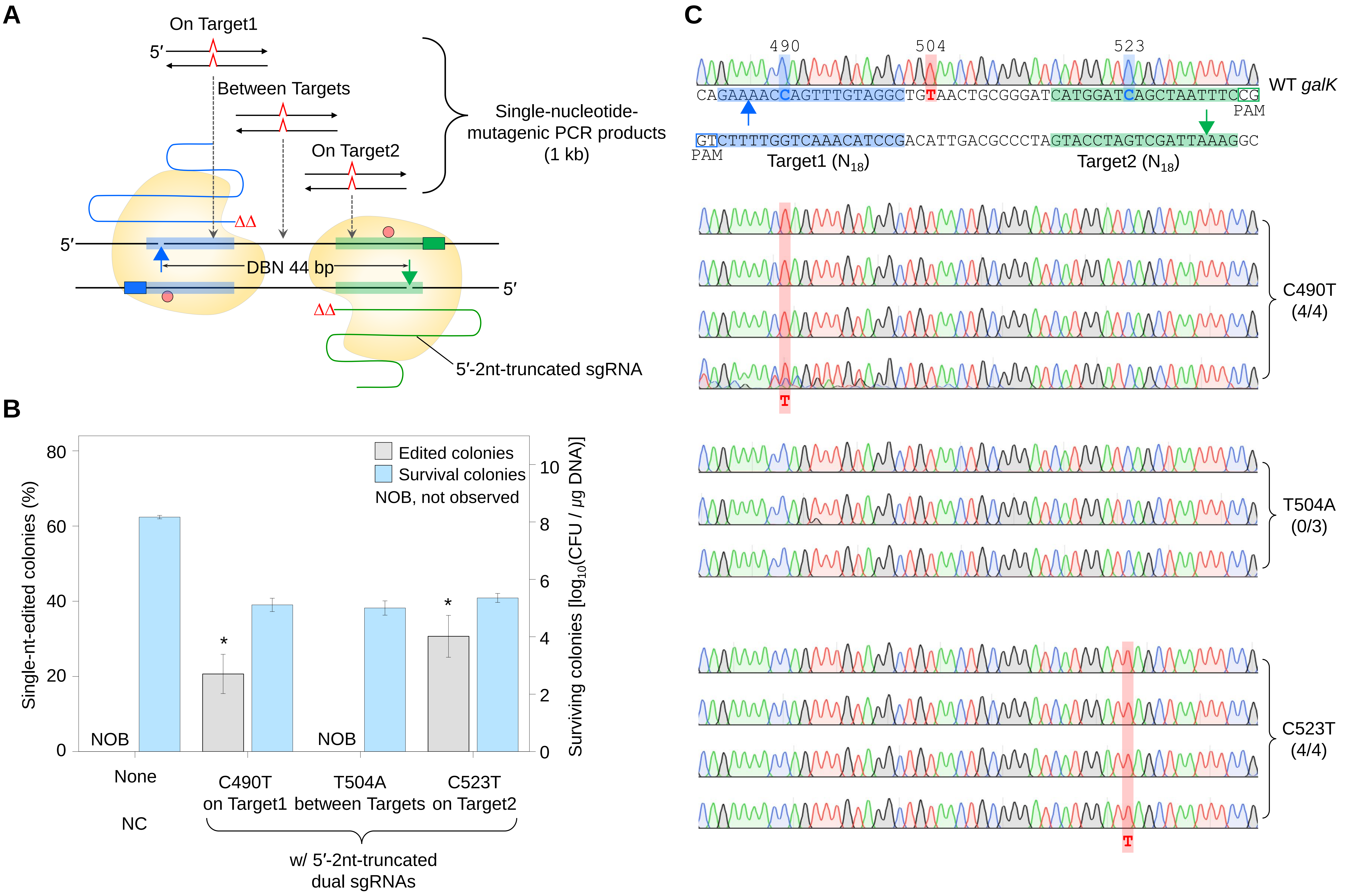


**Supplementary Figure S11.** Cas9-NG nickase-mediated single-nucleotide editing using 5′-truncated dual sgRNAs and PCR products as donor DNA. **(A)** Schematic representation of genome editing using mutagenic PCR products and dual sgRNAs with 2 nt truncation at the 5′-end. **(B)** Efficiency of single-nucleotide editing at various positions in the *galK* gene. Single-nt-edited colonies (%) were calculated as the proportion of correctly edited cells among randomly selected white colonies. *P*-values were determined by comparing the ratio of single-nt-edited colonies in on Target 1 and on Target 2 with those in between Targets. *$P < 0.05$. Negative control (NC) used a dual sgRNA plasmid targeting the *galK* and *xylB* genes. **(C)** Sanger sequences of single-nucleotide-edited *galK* targets. Bold sequences indicate the target nucleotides to be edited. Blue- and green-shaded sequences indicate the target DNA hybridized with dual sgRNAs. Red-shaded chromatograms indicate correctly edited bases. Bold red sequences are intended mutations. Numbers in parentheses are correctly edited colonies among white colonies selected for Sanger sequencing.

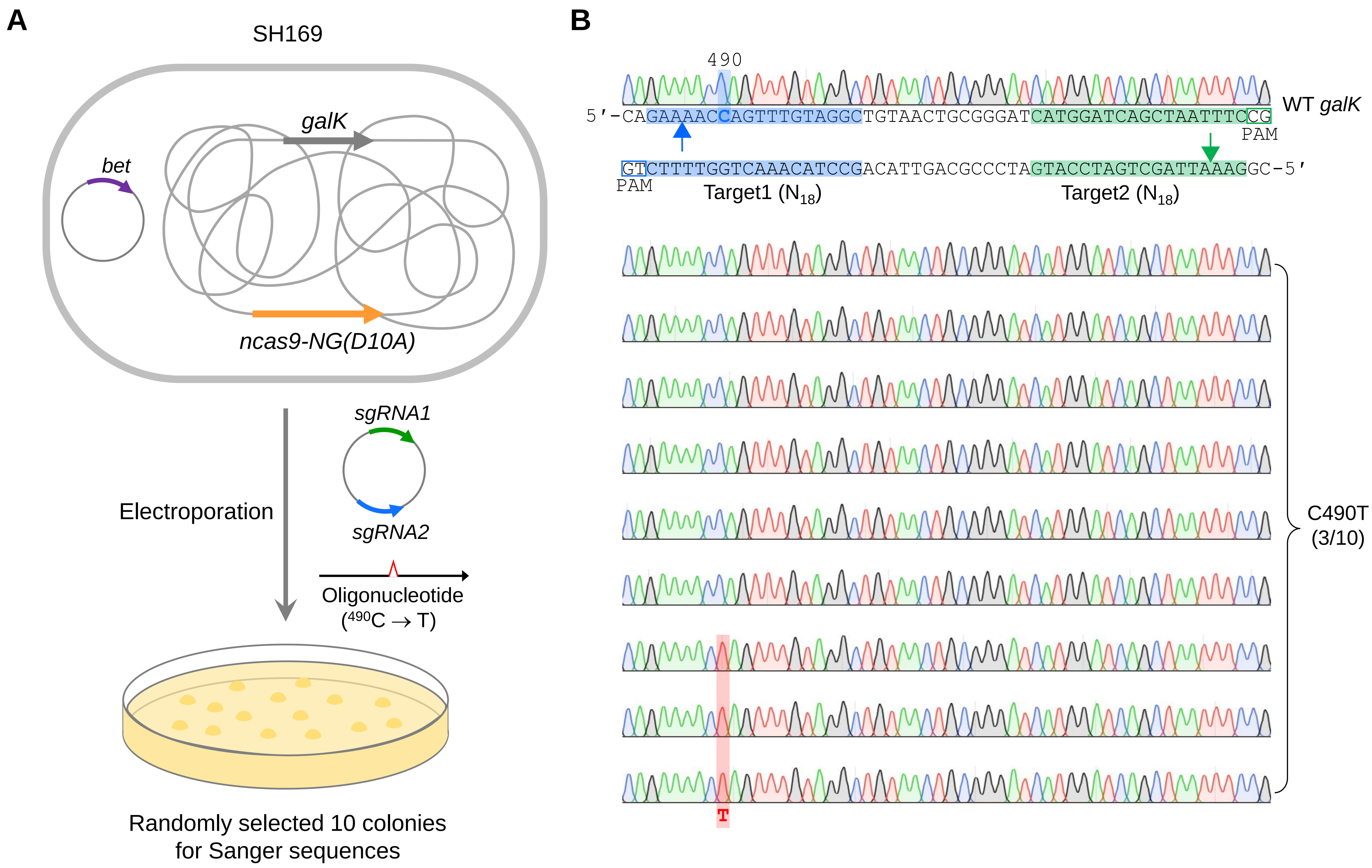


**Supplementary Figure S12.** Single-nucleotide editing using 5′-truncated dual sgRNA without phenotypic change analysis. **(A)** Dual sgRNA plasmid and mutagenic oligonucleotide carrying C490T mutation in the *galK* gene were electroporated into *E. coli* cells overexpressing Cas9-NG nickase and Bet protein. Recovered cells are spread on LB agar plate. **(B)** Sanger sequences of edited *galK* targets in colonies randomly selected from LB agar containing spectinomycin. Blue bold sequence indicate the target nucleotide for single-nucleotide editing. Blue- and green-shaded sequences are the target DNA corresponding to dual sgRNAs. Red-shaded chromatograms indicate correctly edited bases. Altered bases are marked with red bold letters.
